# Multivalent Calixarene-Based Liposomes as Platforms for Gene and Drug Delivery

**DOI:** 10.3390/pharmaceutics13081250

**Published:** 2021-08-12

**Authors:** José Antonio Lebrón, Manuel López-López, Clara B. García-Calderón, Ivan V. Rosado, Fernando R. Balestra, Pablo Huertas, Roman V. Rodik, Vitaly I. Kalchenko, Eva Bernal, María Luisa Moyá, Pilar López-Cornejo, Francisco J. Ostos

**Affiliations:** 1 Department of Physical Chemistry, Faculty of Chemistry, University of Seville, C/Profesor García González 1, 41012 Seville, Spain; jlebron@us.es (J.A.L.); evabernal@us.es (E.B.); 2 Department of Chemical Engineering, Physical Chemistry and Materials Science, Faculty of Experimental Sciences, University of Huelva, Campus de El Carmen, Avda. de las Fuerzas Armadas s/n, 21071 Huelva, Spain; manuel.lopez@diq.uhu.es; 3 Institute of Biomedicine of Seville (IBiS), University Hospital Virgen del Rocío/CSIC/University of Seville, Avda. Manuel Siurot s/n, 41013 Seville, Spain; claragarcia@us.es (C.B.G.-C.); ivrosado@us.es (I.V.R.); 4 Department of Genetics, Faculty of Biology, University of Seville, C/Profesor García González 1, 41012 Seville, Spain; fernando.balestra@cabimer.es (F.R.B.); phuertas@us.es (P.H.); 5 Andalusian Center of Molecular Biology and Regenerative Medicine (CABIMER), University of Seville-CSIC-University Pablo de Olavide, Avda. Américo Vespucio 24, 41092 Seville, Spain; 6 Institute of Organic Chemistry, National Academy of Science of Ukraine, Murmanska Str. 5, 02660 Kiev, Ukraine; dmso@ukr.net (R.V.R.); vik@ioch.kiev.ua (V.I.K.)

**Keywords:** cationic calix[4]arenes, liposomes, nucleic acids, transfection efficiency, doxorubicin, encapsulation

## Abstract

The formation of calixarene-based liposomes was investigated, and the characterization of these nanostructures was carried out using several techniques. Four amphiphilic calixarenes were used. The length of the hydrophobic chains attached to the lower rim as well as the nature of the polar group present in the upper rim of the calixarenes were varied. The lipid bilayer was formed with one calixarene and with the phospholipid 1,2-dioleoyl-sn-glycero-3-phosphoethanolamine, DOPE. The cytotoxicity of the liposomes for various cell lines was also studied. From the results obtained, the liposomes formed with the least cytotoxic calixarene, (TEAC_12_)_4_, were used as nanocarriers of both nucleic acids and the antineoplastic drug doxorubicin, DOX. Results showed that (TEAC_12_)_4_/DOPE/p-EGFP-C1 lipoplexes, of a given composition, can transfect the genetic material, although the transfection efficiency substantially increases in the presence of an additional amount of DOPE as coadjuvant. On the other hand, the (TEAC_12_)_4_/DOPE liposomes present a high doxorubicin encapsulation efficiency, and a slow controlled release, which could diminish the side effects of the drug.

## 1. Introduction

Liposomes are spherical structures, similar to vesicles, which have an inner aqueous polar region and a hydrophobic lipid bilayer [1]. Their spontaneous formation in aqueous solutions is due to interactions among water molecules, hydrophilic head groups and hydrophobic chains of the amphiphilic molecules forming the lipid bilayers [2,3,4,5]. Liposomes have been prepared by several techniques such as thin lipid film hydration, solvent injection, detergent dialysis or reverse phase evaporation [2,4,5]. They are usually characterized according to their size and number of bilayers, and their charge can be positive, negative or neutral [6,7].

Since the pioneering work by Bangham [8], liposomes have been broadly used as delivery systems for several diagnostic and therapeutic compounds including drugs, genes, imaging agents, proteins, or vaccines, among others [9,10,11,12,13]. Liposomes draw great interest for many researchers working on biomedical applications due to the numerous advantages they offer. It is easy to prepare biocompatible and biodegradable liposomes, with low toxicity and immunogenicity, high stability, and with the ability to host hydrophilic and hydrophobic compounds, whose release can be controlled [2,13,14]. A great variety of amphiphilic molecules have been used to prepare liposomes, making the formation of vesicles in aqueous solution favorable [15,16,17]. Among them, calixarenes have been frequently used [18,19,20].

Calixarenes are macrocycles prepared by the base-catalyzed condensation of formaldehyde and p-substituted phenols [21]. They are composed of phenolic units disposed in cyclic arrays and linked by methylene spacers. Their name comes from the word *calix crater* because their tridimensional structure resembles that of an ancient Greek vase. The most common calixarenes are calix[4]arenes, calix[6]arenes, and calix[8]arenes, where [n] indicates the number of phenolic units. The synthetic, easy-to-obtain multivalent ligands by introducing substituents of different nature at the upper and lower rim is one of their main advantages. The structural variety of calixarenes permits to have selective receptors for the inclusion of several neutral molecules, metal and ammonium anions, and cations [22]. Besides, calixarenes can be used in the self-assembly of nanoparticles [23], in the building of molecular machines and rotaxanes [24], for molecular encapsulation [25], and many other applications [22,26,27].

**Scheme 1:**
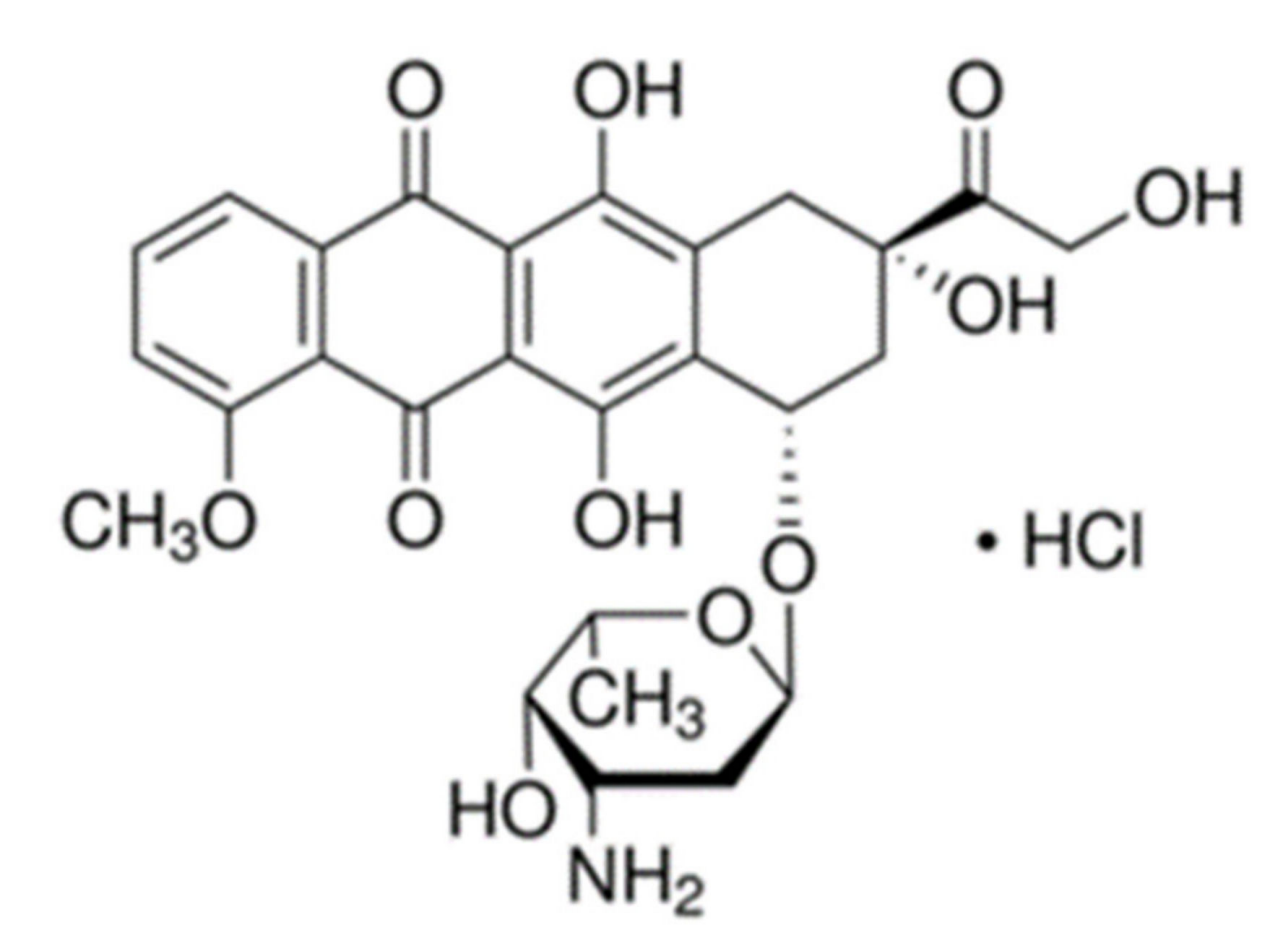
Structure of doxorubicin.

The ability of calixarenes to bind, condense, and transport DNA across cell membranes has been previously investigated [28]. In particular, calix[4]arenes in cone conformation have been found to facilitate cell transfection effectively. Among them, those with long alkyl chains usually lead to small aggregates with low polydispersity, promoting more efficiently gene transfection [29]. On the other hand, it was previously shown that calixarenes interact with the antineoplastic drug doxorubicin, DOX (Scheme 1) [30]. The interaction between calixarenes and DOX is mainly mediated by host–guest and π–π interactions. Works in the literature have shown that calixarenes can be used for the treatment of different types of cancer [31], the results obtained for distinct cancer lines showing that a higher therapeutic effect of the drug is achieved as well as a decrease in side effects.

**Scheme 2:**
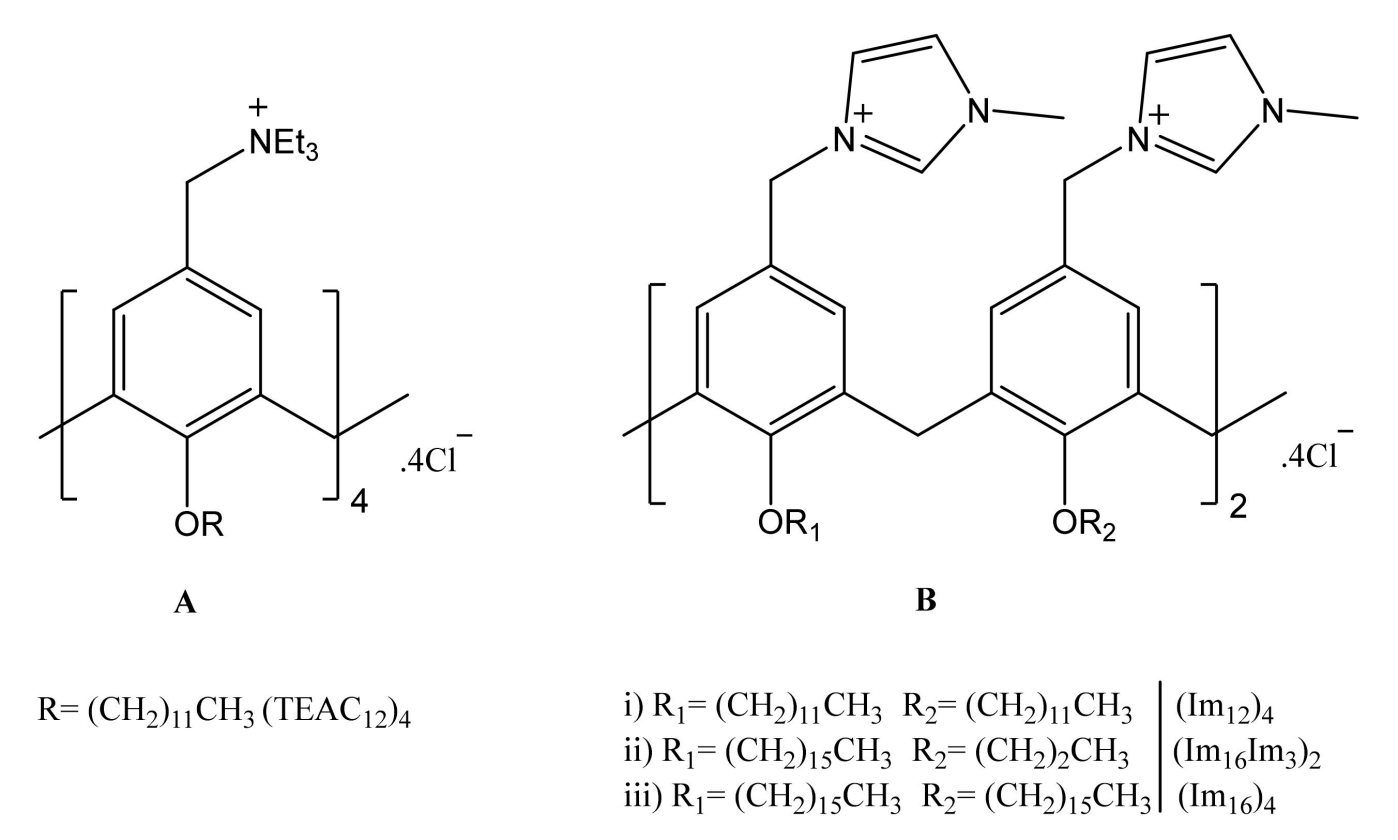
Structure of the calix[4]arenes investigated in this work. (**A**) Calix[4]arene ammonium derivate; and (**B**) Calix[4]arene imidazolinium derivates.

The authors have been interested in the interaction of calixarenes with nucleic acids and antineoplastic drugs such as DOX [30,32,33,34]. In this work, the authors wanted to investigate if the use of calixarene-based liposomes improves the results of their use as nanodelivery systems as compared to the naked calixarenes. It is worth noting that thanks to their unique structural properties, calixarene-based liposomes could provide hybrid systems that will synergistically lead to non-viral vectors with enhanced cell transfection properties [35,36]. Moreover, the hydrophobic cavity of calixarenes provide the posibility that, within the calixarene-based liposomes, host–guest phenomena with different drugs can occur [37]. The more appropriate type of calixarenes for preparing calixarene-based liposomes are the amphiphilic ones. They can be obtained by introducing polar groups at one rim and hydrophobic chains at the other rim [30]. With the goal of preparing calixarene-based liposomes for delivering of genetic material and doxorubicin, in this work the cationic calix[4]arenes 5,11,17,23-tetratriethylammonium-methylene-25,26,27,28- tetradodecyloxycalix[4]arene tetrachloride, (TEAC_12_)_4_; 5,11,17,23-tetra(3-methylimidazolium)-methylene-25,26,27,28-tetradodecyloxycalix[4]arene tetrachloride, (Im_12_)_4_; 5,11,17,23-tetra(3-methylimidazolium)-methylene-25,27-dihexadecyloxy-26,28-dipropoxycalix[4]arene tetrachloride, (Im_16_Im_3_)_2_; and 5,11,17,23-tetra(3-methylimidazolium)-methylene-25,26,27,28-tetrahexadecyloxycalix[4]arene tetrachloride, (Im_16_)_4_, have been used to prepared liposomes (see Scheme 2). The abbreviations used tried to inform the reader about the structure of the calixarenes. TEAC stands for tetraethylammonium chloride and Im for imidazolinium, in order to distinguish between the two different charged heads present in the upper rim of the calixarenes. The subscripts 3, 12, and 16 inside the parentheses indicate the length of the hydrophobic chains attached to the lower rim. The subscripts 2 and 4 outside the parentheses indicate the number of units inside the parentheses present in the calixarene molecules. Throughout this work the abbreviation CAL means calixarene.

The results obtained will be of interest for researchers working on the use of calixarenes in several biotechnological applications.

## 2. Materials and Methods

### 2.1. Materials

The lipid 1,2-dioleoyl-sn-glycero-3-phosphoethanolamine (DOPE) was purchased from Avanti Polar Lipids (Alabaster, AL, USA). Red Safe was from iNtRON (Biotechnologiy, Chicago, IL, USA). The rest of the materials, including doxorubicin, were from Sigma-Aldrich (Darmstadt, Germany) and used without further purification.

ctDNA concentration was estimated by UV–visible spectroscopy measuring at 260 nm (molar absorptivity 6600 mol^−1^ dm^3^ cm^−1^ [38]). The average number of base pairs was estimated by agarose gel electrophoresis, using ethidium bromide, EB. The results indicate that there are above 10,000 bp [39]. Throughout the manuscript, the ctDNA concentration will be expressed per base-pairs. The pEGFP-C1 plasmid (Clontech, Biocientífica S.A., Buenos Aires, Argentina), pDNA, was extracted from competent *E*. *coli* bacteria previously transformed with pEGFP-C1; the extraction was done using a GenElute HP Select Plasmid Gigaprep kit (Sigma Aldrich, Darmstadt, Germany). A protocol previously described was used [40]. FuGENE 6 was from Promega Corporation (Madison, WI, USA).

The syntheses of the cationic calixarenes (TEAC_12_)_4_ and (Im_16_Im_3_)_2_ were previously described [30], and (Im_12_)_4_ and (Im_16_)_4_ were purchased from Life Chemicals Inc. (Niagara-on-the-Lake, ON, Canada). Their purity (≥99%) was checked by ^1^H and ^13^C NMR, elemental analysis, and mass spectra.

Solutions were prepared with MilliQ water (resistivity > 18 MΩ × cm). The pH was kept constant at 7.4 by using 10 mM HEPES (4-(2-hydroxyethyl)piperazine-1-ethanesulfonic acid sodium salt) buffer.

### 2.2. Preparation of Liposomes

Liposomes were prepared using the lipid thin-film hydration method [41]. Briefly, adequate quantities of calixarenes, CAL, and DOPE were dissolved in chloroform. Different volumes of these solutions were mixed in order to obtain the desired cationic calixarene molar fraction, α, given by:

(1) $$\alpha =\frac{n_{\mathrm{CAL}}}{n_{\mathrm{CAL}}+n_{\mathrm{DOPE}}}$$

where $n_{\mathrm{CAL}}$ and $n_{\mathrm{DOPE}}$ are the mole number of the cationic calixarene and the zwitterionic DOPE, respectively, in the total volume of the organic solution.

A rotary evaporator was used to evaporate the organic solvent, at 303 K for 50 min. The resultant dry lipid film was stored at 193 K for at least 24 h. In this way, degradation is avoided [42]. Afterwards, 2 mL of HEPES 10 mM, pH = 7.4, was added for hydrating the lipid film, and the mixture was submitted to 10 cycles of vortex (3 min/1200 rpm) and sonication (2 min, JP Selecta Ultrasons system 200 W, 50 kHz, Abrera, Barcelona, Spain). In the final step the solution was vortexed for 2 h at room temperature. The liposome solution had a high polydispersity, with multilamellar liposomes. In order to obtain a homogeneous size distribution solution with unilamellar liposomes, 1 mL of liposome solution was extruded 10 times with a manual mini extruder from Avanti Polar Lipids (Alabaster, AL, USA), using polycarbonate membranes of 100 and 200 nm (Whatman, Maidstone, UK). After extrusion, the solutions were maintained in the dark at 277 K for 24 h for a complete stabilization. In this work the calixarene/DOPE liposomes will be named CAL/DOPE liposomes.

Only in the case of (Im_16_)_4_ liposomes was a mixture of ethyl acetate:ethanol 1:1 used, instead of chloroform, because of solubility problems. Nonetheless, by using this mixture in the thin film hydration method, the characteristics of the liposomes containing (TEAC_12_)_4_, (Im_12_)_4_, and (Im_3_Im_16_)_2_ were similar to those observed using chloroform.

The composition (mole ratio) of the liposomes prepared is summarized in Table 1.

### 2.3. Preparation of Lipoplexes

The lipoplexes were prepared by mixing appropriate volumes of the liposome solution and of the aqueous ctDNA (or p-EFGP-C1) HEPES 10 mM solutions in order to obtain the desired L/D ratio. For each α value, the mass ratio L/D is given by the expression:

(2) $$\frac{L}{D}=\frac{m_{\mathrm{CAL}}+m_{\mathrm{DOPE}}}{m_{\mathrm{DNA}}}$$

where $m_{\mathrm{DOPE}}$, $m_{\mathrm{CAL}}$, and $m_{\mathrm{DNA}}$ are the masses of the zwitterionic phospholipid, of the calixarene, and of the DNA, respectively, in the solution. In all the liposome solutions investigated, the mass of DNA was kept constant at 10^−4^ g (the concentration was 1.0 mg/mL or 8.1 × 10^−5^ mol L^−1^ given in base-pairs). The calixarene/DOPE/ctDNA lipoplexes will be named CAL/DOPE/DNA lipoplexes.

The stability of the lipoplexes was followed by changes in their size and polydispersity with time. The size remained unchanged for more than 48 h. The authors also checked the stability of the lipoplexes, of different compositions, after dilution with buffer HEPES 10 mM. No variations in their size were observed.

### 2.4. Zeta Potential Measurements

Zeta-potential, ζ, values were calculated measuring the electrophoretic mobility of the liposomes and of the lipoplexes from the velocity of the particles, using a laser Doppler velocimeter (LDV). A Zetasizer Nano ZS Malvern Instrument Ltd. (Malvern, Worcestershire, UK) was used. Temperature was kept at 303.0 ± 0.1 K, and DTS1060 polycarbonatecapillary cells were utilized. ctDNA concentration in the buffered solutions of liposomes was 8.1 × 10^−5^ M. Data are expressed as mean ± SD from at least three separate experiments, *n* = 9.

### 2.5. Dynamic Light Scattering, DLS, Measurements

A Zetasizer Nano ZS Malvern Instrument Ltd. (Worcestershire, UK) was used to estimate the hydrodynamic diameter, d_H_ (Z average), and the polydispersity index, PDI, of the lipoplexes using DLS measurements. A scattering angle of 90° was used. A fixed concentration of 8.1 × 10^−5^ M of ctDNA was present in all the liposome solutions investigated. Data are expressed as mean ± SD from at least three separate experiments, *n* = 9. Temperature was maintained at 303.0 ± 0.1 K.

### 2.6. Agarose Gel Electrophoresis

Agarose gel (1%) was prepared in a TAE buffer (40 mM Tris-acetate, 1 mM EDTA) in a total volume of 180 µL and stained with the dye Red Safe (10 μL) for the visualization of the nucleic acid bands. The ctDNA concentration was kept constant at 8.1 × 10^−5^ M. The method was as follows: (i) 20 μL of the buffered liposome solution was mixed with 5 μL of 5 × DNA loading buffer. (ii) After homogenization, the resulting solution was added in each well. Electrophoresis was performed at 90 V for 90 min. A detector Ultima 16si (Hoefer Inc., Holliston, MA, USA) was used for visualizing the nucleic acid bands by irradiation with UV light (254 nm).

### 2.7. Circular Dichroism, CD, Spectra

A Biologic Mos-450 spectropolarimeter (Cambridge, UK) was used to register the CD spectra. Scans were taken from 220 to 310 nm with a standard quartz cell of 10 mm path length. Three independent experiments were done. Each spectrum was obtained from an average of 10 runs, with a 5 min equilibration before each scan, at 303.0 ± 0.1 K. The ctDNA concentration was kept constant at 8.1 × 10^−5^ M in the lipoplex solutions. All solutions were prepared in 10 mM HEPES buffer, pH = 7.4.

### 2.8. Atomic Force Microscopy, AFM

Atomic force microscopy was used to study the structures of the lipoplexes. A resonance frequency of around 240 KHz and a nominal force constant of 42 N/m were the working conditions in a Molecular Imaging PicoPlus 2500 AFM (Agilent Technologies, Santa Clara, CA, USA). Silicon cantilevers (Model Pointprobe, Nanoworld, Neufchâtel, Switzerland) were used. The images were recorded in air and in tapping mode. Data collection (256 × 256 pixels) was registered with scan speeds about 0.5 Hz. The ctDNA concentration in the buffered HEPES 10 mM liposome solutions, pH = 7.4, was 0.6 μM.

Images of the buffered liposome solutions were obtained using the following method: (a) In order to prepare a modified mica surface, 0.1% (*v*/*v*) APTES aqueous solution was dropped onto a freshly cleaved mica surface. It was washed with ultra-pure water after 20 min and air dried. (b) A 30 μL droplet of the lipoplex solution was deposited on the modified mica surface and incubated for 30 min. (c) Subsequently, the mica surface was washed with pure water and air dried for AFM imaging.

### 2.9. Electron Transmission Microscopy, TEM

TEM images of the lipoplexes were obtained in a Zeiss Libra 120 scanning electron microscope (Carl Zeiss AG, Oberkochen, Germany), at 80 kV. Samples were prepared by impregnation, using a 300 mesh copper grid coated collodion that, subsequently, was stained with a solution of uranyl acetate (2.0%). Images were processed with a bottom-mounted TEM CCD camera and recorded with a resolution of 2048 × 2048 pixels. ImageJ (National Institutes of Health (NIH), Bethesda, MD, USA) bundled with 64-bit Java 1.8.0_172 was used to analyze TEM images from independent experiments, for each of the lipoplex solutions investigated.

### 2.10. In Vitro Cytotoxicity Assays

The cytotoxicity of the CAL/DOPE liposomes with α = 0.5 for (TEAC_12_)_4_, (Im_12_)_4_, and (Im_16_Im_3_)_2_, and with α = 0.3 for (Im_16_)_4_, at different L/D values, was estimated in vitro using the MTT assay [43]. These are the maximum α values that could be prepared for the different calixarenes, and they were chosen to carry out the cell viability assays because they correspond to the highest content of the cationic calixarene within the liposomes. The cell lines used were RPE-1 (normal cell line), A549 (adenocarcinomic human alveolar basal epithelial cell line), HepG2 (human liver cancer cell line), LS180 (adenocarcinomic human colonic epithelial cell line), and MCF7 (breast cancer cell line). They were a gift from different research groups from the IBIS (the Institute of Biomedicine of Seville). In any case, all cell lines used were from commercial suppliers. Cell lines were plated out into 96-well plates at a density of 3000 cells per plate. The next day, the liposome solutions were added to the wells, and the plate wa s returned to the incubator for 4 days more. Later, they were pulsed with MTS (ROCHE, Basilea, Switzerland). According to the manufacturer instructions, cell viability was measured by luminometry in a Varioskan Flash (Thermo Fisher Scientific, Waltham, MA, USA). Each liposome concentration was measured in triplicate.

### 2.11. Transfection Assays

The cell line chosen to carry out these experiments was the U2OS, from human osteosarcoma, because these cells are suitable for transfection experiments. The non-viral vectors investigated were (TEAC_12_)_4_/DOPE liposomes, containing the plasmid pEGFP-C1. Liposomes of (TEAC_12_)_4_ were selected because they are the least toxic of all the CAL/DOPE liposomes investigated. On the other hand, pEGFP-C1 is a plasmid carrying an enhanced GFP coding sequence with the required regulatory elements for constitutive expression of the gene in human cells. The method used to carry out the transfection experiments was as follows: 3 μg of pEGFP-C1 was added to a solution containing 180 μL of Opti-MEM (Gibco, Thermo Scientific, Waltham, MA, USA), and the amount of liposome buffered solution (HEPES 10 mM) necessary to obtain the L/D ratio for each α value was investigated. The resulting mixture was incubated at room temperature for 20 min, and afterwards it was added to a 50% confluent 6 cm plate with 3 mL of DMEM medium (Sigma Aldrich, Darmstadt, Germany).

The cells were transfected with a mixture of transfection reagent and Opti-MEM (not pEGFP-C1 included) as negative control. As positive control, FuGENE 6 transfection reagent (E2311, from Promega Corporation, Madison, WI, USA) was used, according to the manufacturer’s protocol (i.e., 3 μg of pEGFP-C1 in 200 μL Opti-MEM plus 9 μL of FuGENE 6). Transfection efficiency was evaluated by flow cytometry with a FACSCalibur (BD Biosciences, Franklin Lakes, NJ, USA) 24 h after transfection.

### 2.12. UV–Visible Spectroscopy

The doxorubicin concentration was determined by UV–visible spectroscopy, using a Hitachi UV-visible 3900 (Chiyoda, Tokyo, Japan) by measuring absorbance at 490 nm. Temperature was kept using a Lauda (Stuttgart, Baden-Würtenberg, Germany) flow cryostat connected to the cell compartment.

### 2.13. Encapsulation Efficiency Measurements

A dialysis method was used to estimate the doxorubicin, DOX, encapsulation efficiency of the (TEAC_12_)_4_/DOPE liposomes. A total of 900 μL of drug-loaded liposomes was added to a Spectra/Por^®^ 3 (MWCO 3.5 kDa) from Spectrum Laboratories, Inc. (Rancho Dominguez, CA, USA) The final concentration of antibiotic was 2 × 10^−4^ M. The dialysis membrane was plunged into a beaker containing 30 mL of 10 mM HEPES buffer (pH = 7.4), the same used for liposome hydration. The liposomes’ stabilization was ensured by keeping the temperature at 4 °C throughout the process in order to avoid doxorubicin degradation [32]. An aliquot of 1 mL from the beaker was taken at different time intervals. These aliquots were replaced each time by an equal volume of buffer HEPES 10 mM in order to keep constant the total volume of buffer in the beaker. Dialysis was followed for at least 24 h. The encapsulation efficiency, EE, was calculated by using Equation (3):

(3) $$\mathrm{EE}\left(\%\right)=\frac{{\left\lceil \mathrm{DOX}\right\rceil }_{\mathrm{Liposomes}}}{{\left[\mathrm{DOX}\right]}_{T}}\times 100=\frac{{\left[\mathrm{DOX}\right]}_{T}-{\left[\mathrm{DOX}\right]}_{\mathrm{buffer}}}{{\left[\mathrm{DOX}\right]}_{T}}$$

where ${\left\lceil \mathrm{DOX}\right\rceil }_{\mathrm{Liposomes}}$, ${\left[\mathrm{DOX}\right]}_{T}$, and ${\left[\mathrm{DOX}\right]}_{\mathrm{buffer}}$ are the DOX concentration encapsulated into the calixarene-based liposomes, the total DOX concentration present in the system, and the DOX concentration in the buffer solution, respectively. All the concentrations are referred to the total volume of solutions.

The loading capacity, LC, can be calculated using the following expression:

(4) $$\mathrm{LC}=\frac{n_{\mathrm{DOX}.\mathrm{enc}.}}{n_{\mathrm{DOX},\mathrm{total}}+n_{\mathrm{CAL}+\mathrm{DOPE}}}\times 100$$

where $n_{\mathrm{DOX}.\mathrm{enc}.}$, $n_{\mathrm{DOX},\mathrm{total}}$, and $n_{\mathrm{CAL}+\mathrm{DOPE}}$ represent the moles of the encapsulated drug, total drug, and total lipids, respectively.

Quantification of doxorubicin concentration was done by UV–visible spectroscopy (λ = 490 nm). Each experiment was performed in triplicate.

### 2.14. In Vitro Drug Release

After applying the dyalisis method, the doxorubicin loaded (TEAC_12_)_4_/DOPE liposomes were suspended in buffer HEPES 10 mM, pH = 7.4, in a glass vial under continuous magnetic stirring, 200 rpm, at 37.4 °C (the human body temperature). A sample was removed at determined time intervals and, subsequently, replaced with an equal amount of buffer. This is a way to simulate the in vivo removal of a drug into a systemic circulation. The concentration of the antibiotic was estimated by UV–visible spectroscopy, measuring the absorbance at 490 nm. The absorbance data were corrected from the dilution effect. Three separate experiments were done. The precision was close to 7%.

### 2.15. Statistical Analysis

Values are expressed as the mean ± standard errors of independent experiments. Statistical analysis was performed with Student’s t-test and one-way analysis of variance (ANOVA). When *p* < 0.05 (95% confidence) the differences were considered as significant.

## 3. Results and Discussion

### 3.1. Calixarene-Based Liposomes

**Figure 1:**
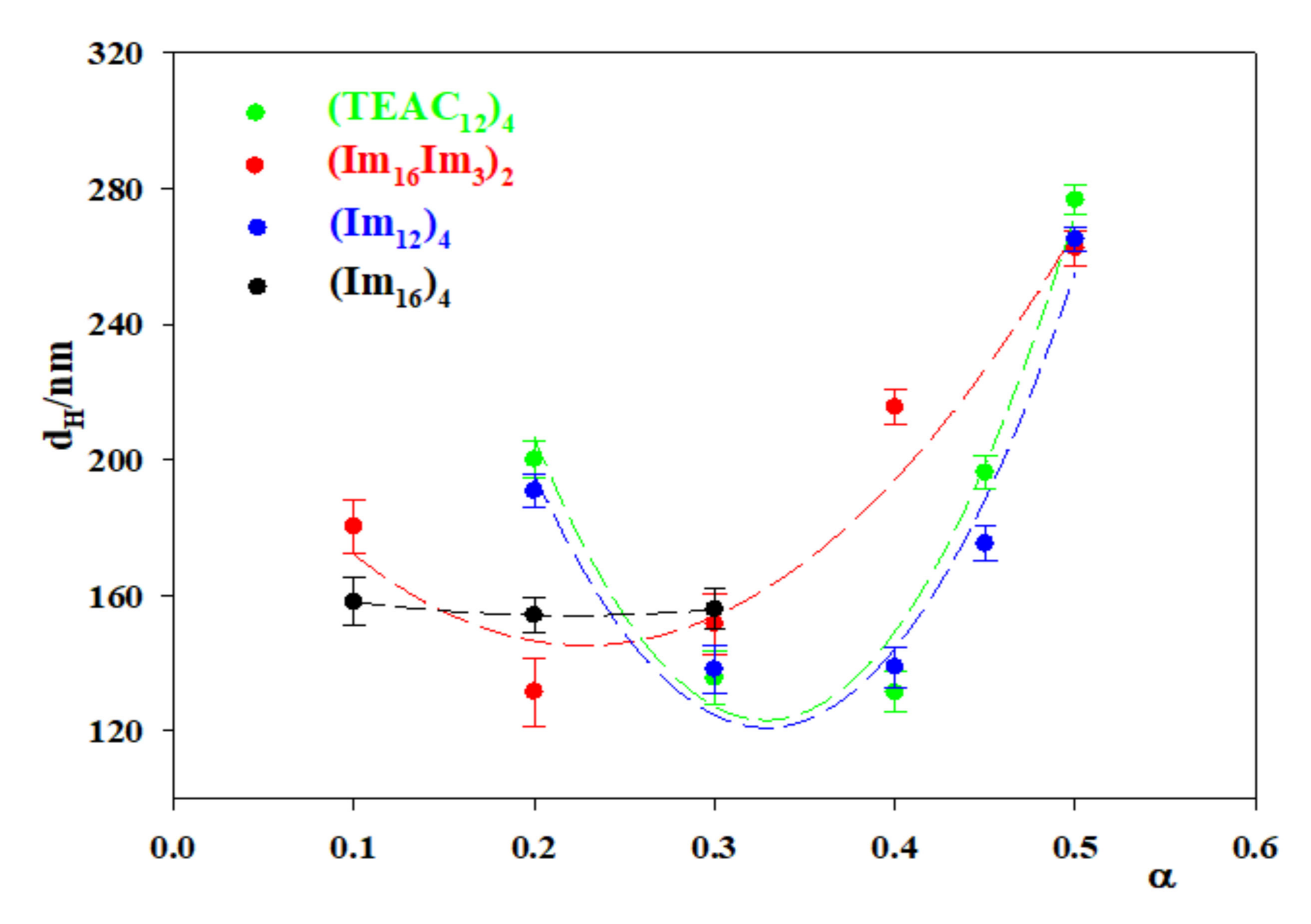
Dependence of the hydrodynamic diameter, d_H_, on the cationic calixarene molar fraction, α. T = 303.1 ± 0.1 K.

Calixarene-based liposomes were prepared at several cationic CAL molar fractions α (α = n_CAL_/(n_CAL_+ n_DOPE_)). In particular, this magnitude was varied within the interval from 0.1 to 0.5. Molar ratios higher than 0.5 were not investigated because these liposome solutions showed a high polydispersity index, PDI (PDI > 0.8). Figure 1 shows the dependence of the hydrodynamic diameter of the calixarene-based liposomes on the calixarene molar ratio, α, in these nanostructures. The interval of α studied for each calixarene was limited by solubility problems. In the case of (Im_16_)_4_, it was particularly narrow. Figure 1 shows that the size of the CAL/DOPE liposomes initially decreased upon increasing α, but a subsequent increase in the molar ratio led to an increase in the liposome sizes. This trend was observed for all CAL/DOPE liposomes with the exception of (Im_16_)_4_, for which no clear trend was observed due to the narrow α interval studied.

The experimental observations could be explained by considering the main interactions controlling the liposome sizes. On one hand the hydrophobic interactions are the driving force for the formation of liposomes, and they favor a diminution in the liposome size [44]. On the other hand, the electrostatic repulsions between the positively charged head groups of the calixarenes in the bilayer make an increase in the liposome size more favorable. At low α values the hydrophobic interactions mainly control the liposome sizes, and an increment in α results in a diminution of the hydrodynamic diameter, d_H_. A further increment in α means an increase in the amount of cationic CAL within the liposomes, and, as a consequence, the electrostatic repulsions will augment. At a given α value, depending on the calixarene nature, the electrostatic repulsions overcome the hydrophobic interactions, and an increase in α is followed by an increase in d_H_ since the cationic head groups tend to separate. The hydrophobic interactions are expected to be stronger in the case of (TEAC_12_)_4_ and (Im_12_)_4_ than for (Im_3_Im_16_)_2_. The latter has alternate chains, which makes the interaction between the two 16 C tails more difficult. Besides, two of the chains are short (three C atoms), this resulting in weak hydrophobic interactions when compared to those with 12 C atoms. Therefore, the minimum in the plots of d_H_ vs. α is expected to be observed for higher α values in the case of (TEAC_12_)_4_ and (Im_12_)_4_ than in the case of (Im_3_Im_16_)_2_, as in fact is observed. Following the same reasoning, (Im_16)_)_4_ should present the minimum at the highest α value. However, this is not observed.

Apart from the solubility problems, a possible explanation would be that the long hexadecyl chains could fold towards themselves, resulting in steric hindrance [45], which would make smaller liposomes more favorable.

**Figure 2:**
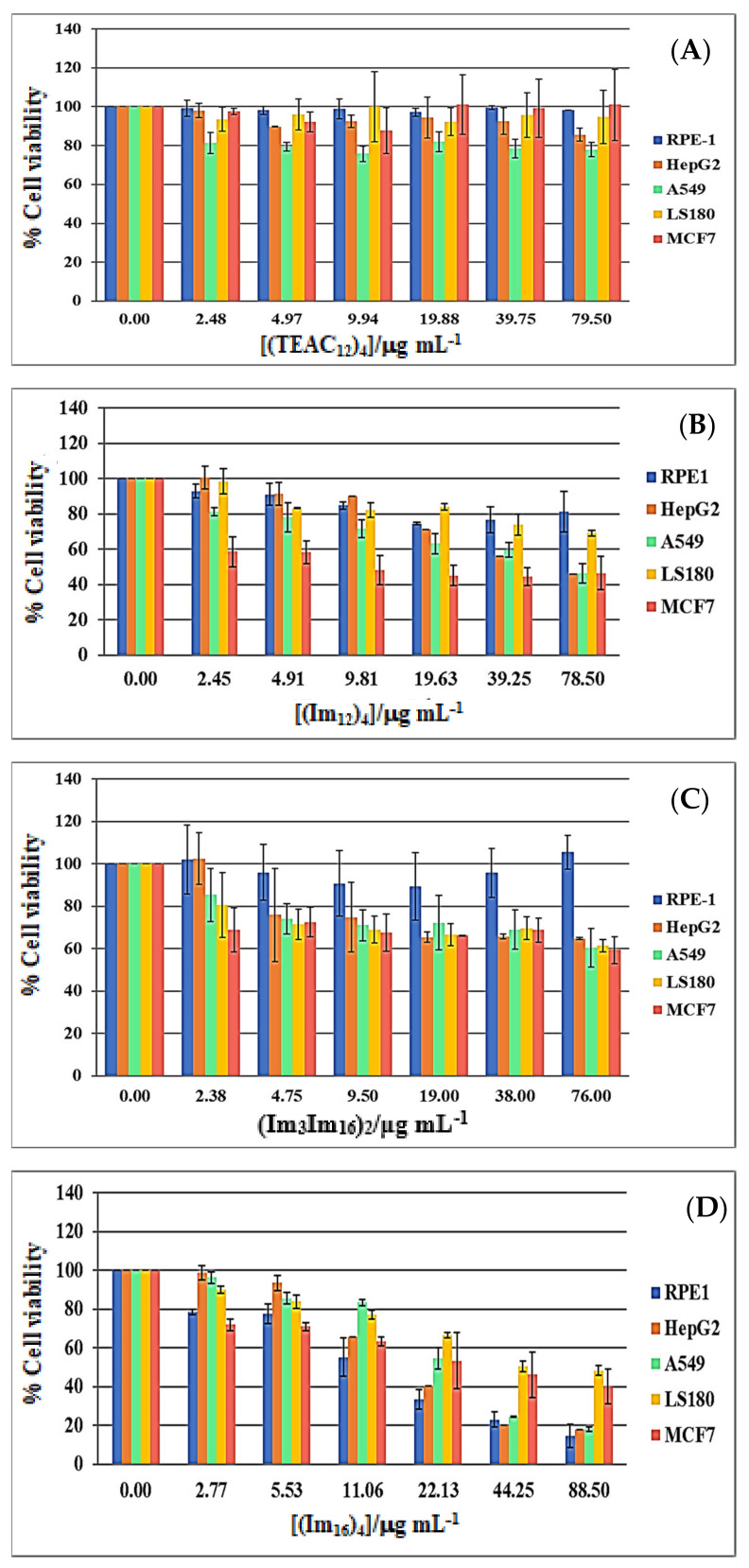
Cell viability values in the presence of different liposome concentrations at α = 0.5 for (TEAC_12_)_4,_ (Im_12_)_4_, and (Im_3_Im_16_)_2_, and at α = 0.3 for (Im_16)_)_4._ The results are the average of three independent experiments: (**A**) (TEAC_12_)_4_; (**B**) (Im_12_)_4_; (**C**) (Im_3_Im_16_)_2_; (**D**) (Im_16_)_4_.

The cytotoxicity of the CAL/DOPE liposomes was estimated by using the MTT assay. Since DOPE is considered a biocompatible lipid, the cell viability in several cell lines was determined for the highest α value, corresponding to the liposomes with the largest amount of cationic calixarenes. Figure 2 shows the results obtained, from which some conclusions can be reached. (TEAC_12_)_4_/DOPE liposomes are the less toxic of all. From the comparison of the data corresponding to the (TEAC_12_)_4_/DOPE liposomes to those of (Im_12_)_4_, one can say that the substitution of quaternary ammonium groups by imidazolinium ones results in an increase in the cytotoxicity of the liposomes. A similar result was observed by Rodik et al. in a previous work [46]. On the other hand, an increase in the length of the hydrophobic chains attached to the lower rim of the calixarenes caused a diminution in the cell viability. This was particularly evident for the (Im_16_)_4_/DOPE liposomes. This behavior can be attributed to a higher lipophilicity, which results in a higher ability to penetrate and disrupt the cell membrane. It was particularly evident for the (Im_16_)_4_/DOPE liposomes. A similar trend has been previously observed by other authors [47]. Besides, Figure 2 shows that (Im_3_Im_16_)_2_/DOPE liposomes present a specific selectivity towards tumor cells, while (Im_12_)_4_/DOPE liposomes act particularly on MCF7 mammary cells and, at high concentrations, on HepG2 and A549 cells.

### 3.2. CAL/DOPE/DNA Lipoplexes

**Figure 3:**
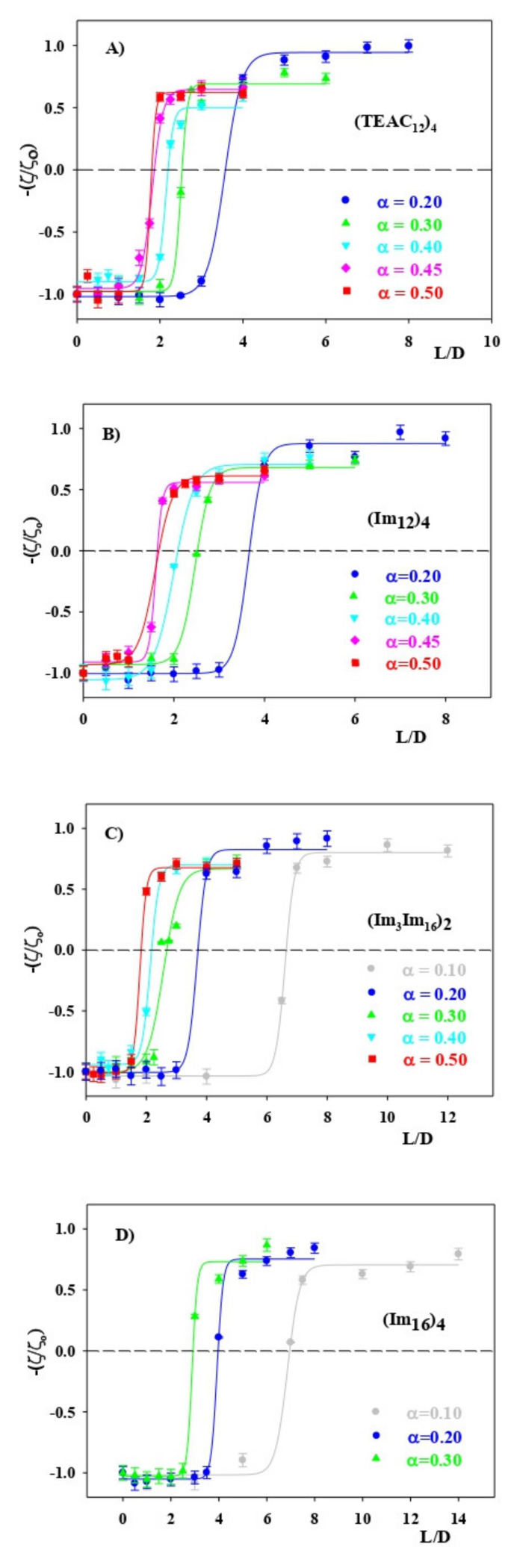
Dependence of the relative zeta potential, −(ζ/ζ_o_), of the CAL/DOPE/DNA lipoplexes on L/D for different molar ratios α. T = 303.0.1 ± 0.1 K. (**A**) (TEAC_12_)_4_; (**B**) (Im_12_)_4_; (**C**) (Im_3_Im_16_)_2_; (**D**) (Im_16_)_4_.

In order to study the characteristics of the CAL/DOPE/DNA lipoplexes, the zeta potential, ζ, was measured for a given molar ratio, α, at different mass ratios L/D. Figure 3 shows the results obtained for the different calixarenes studied in this work. In all cases when L/D increases, the charge of the lipoplex goes from negative to positive. The solutions were stable, and no turbidity was observed in any system. This is an expected result since, for a given α, an increase in L/D means an increment in the amount of cationic lipid, CAL, in the liposomes. The charge inversion observed indicates that the polynucleotide interacts with the cationic calixarene within the lipoplex. From the data in Figure 3 it is possible to calculate (L/D)_Φ_, the value of the mass ratio corresponding to a zeta potential equal to zero; that is, when the lipoplexes are neutral. (L/D)_Φ_ values can also be calculated theoretically using Equation (5). The deduction of this equation is described in the Supplementary Information.

(5) $${\left(\frac{L}{D}\right)}_{\Phi }=\frac{q_{\mathrm{ADN}}^{-}}{q_{\mathrm{CAL}}^{+}}\times \frac{M_{\mathrm{CAL}}}{M_{\mathrm{bp}}}\times \frac{\left(n_{\mathrm{CAL}}M_{\mathrm{CAL}}+{\;n}_{\mathrm{DOPE}}M_{\mathrm{DOPE}}\right)\;}{\left(n_{\mathrm{CAL}}+n_{\mathrm{DOPE}}\right)\;}\times \frac{1}{\frac{n_{\mathrm{CAL}}}{\left(n_{\mathrm{CAL}}+n_{\mathrm{DOPE}}\right)}}=\frac{q_{\mathrm{ADN}}^{-}}{q_{\mathrm{CAL}}^{+}}\times \frac{M_{\mathrm{CAL}}}{M_{\mathrm{bp}}}\times \frac{{\alpha M}_{\mathrm{CAL}}+\left(1-\alpha \right)M_{\mathrm{DOPE}}}{\alpha }$$

where $q_{\mathrm{ADN}}^{-}$ and $q_{\mathrm{CAL}}^{+}$ are the charges of the polynucleotide and of the CAL, respectively. The charge of calf thymus DNA is considered to be−2 per base-pairs [48]. $M_{\mathrm{CAL}}$ and $M_{\mathrm{DOPE}}$ are the molecular weights of the cationic calixarene and non-ionic lipid, respectively, M_bp_ being the polynucleotide molecular weight per base-pair. $n_{\mathrm{CAL}}$ and $n_{\mathrm{DOPE}}$ are the number of moles the CAL and DOPE, respectively. The rest of the symbols have been previously defined. Table 2 summarizes the experimental and the theoretical (L/D)_Φ_ values. One can see that, within experimental errors, the theoretical and the experimental values agree quite well. It is important to know the charge of the different lipoplexes prepared because one of the requirements for an efficient cellular uptake is that the charge of the nanocarrier (the lipoplexes in this work) has to be positive in order to cross the negatively charged cellular membrane [49].

Charge inversion of DNA in the lipoplexes can also be investigated by gel electrophoresis. Figure S1 (Supplementary Information) shows the results obtained by using this technique. In this figure a migration to the anode is observed for L/D values lower than (L/D)_Φ_, for the systems investigated, although a diminution in the mobility of the band is found when L/D approximated to (L/D)_Φ_. Once this value is reached, the mobility is hindered, this pointing out that the charge of the polynucleotide has been inverted from negative to positive. These results are in agreement to those found by zeta potential measurements (see Figure 3).

**Figure 4:**
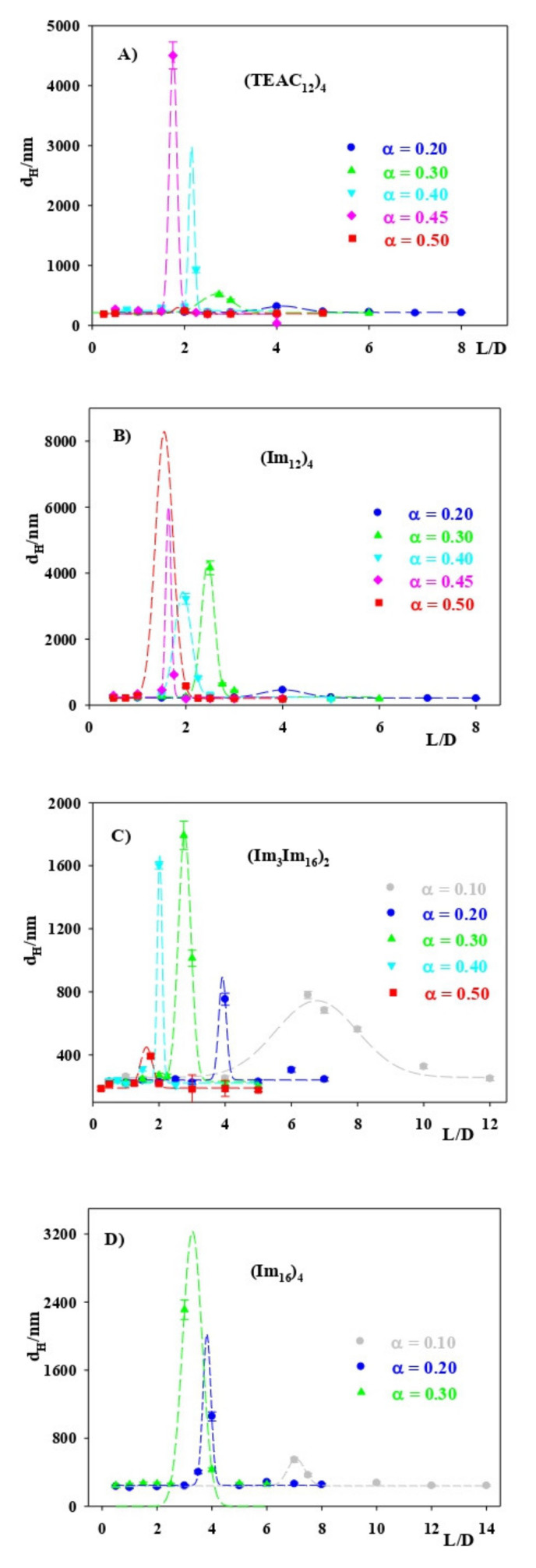
Dependence of the hydrodynamic diameter, d_H_, of the CAL/DOPE/DNA lipoplexes on L/D for different molar ratios α. T = 303 ± 0.1 K. (**A**) (TEAC_12_)_4_; (**B**) (Im_12_)_4_; (**C**) (Im_3_Im_16_)_2_; (**D**) (Im_16_)_4_.

Another important magnitude is the size of the lipoplexes. Recent studies indicated that there is a particular size range adequate for cellular uptake [18,50]. Although cellular uptake depends on the type of cells and on the different barriers making this process difficult [49], the appropriate nanocarrier size is usually considered to be a few hundred nanometers. Figure 4 shows the dependence of the hydrodynamic diameter, d_H_, on the mass ratio L/D, for a given α value, for the calixarenes investigated. In all cases a Gaussian dependence of d_H_ on L/D was observed. This dependence can be explained as follows. At low as well as at high L/D values, the lipoplexes are negatively and positively charged, respectively (see Figure 3). That is, there are repulsive forces among them that kept them apart and maintained a stable size distribution. When L/D is approaching (L/D)_Φ_, the charge of the lipoplexes is moving closer to zero. As a consequence, the lipoplexes do not repel each other and an aggregation process occurs, a steep increment in d_H_ being observed. This explanation is supported by Figure S2 (Supplementary Material), which shows the dependence of the relative zeta potential, (ζ/ζ_o_), and of the hydrodynamic diameter, d_H_, of CAL/DOPE/DNA lipoplexes on L/D for α = 0.2. Apart from the L/D values close to (L/D)_Φ_, the lipoplex sizes observed for the different molar ratios α, and for all the CAL studied, are within the hundred nanometers size range.

**Figure 5:**
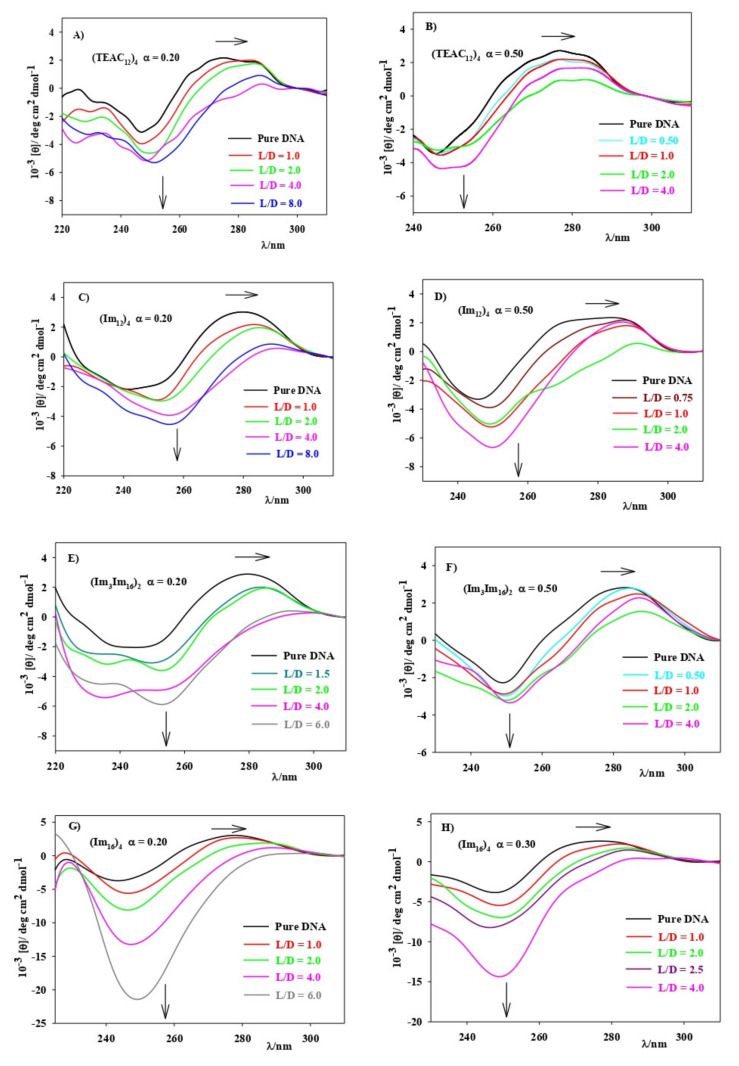
Dependence of the circular dichroism spectra of the CAL/DOPE/DNA lipoplexes on L/D for different molar ratios α. T = 303 ± 0.1 K.; (**A**) α= 0.20 for (TEAC_12_)_4_; (**B**) α = 0.50 for (TEAC_12_)_4_; (**C**) α = 0.20 for (Im_12_)_4_; (**D**) α = 0.50 for (Im_12_)_4_; (**E**) α = 0.20 for (Im_3_Im_16_)_2_; (**F**) α= 0.50 for (Im_3_Im_16_)_2_; (**G**) α = 0.20 for (Im_16_)_4_; and (**H**) α = 0.30 for (Im_16_)_4_.

A way of getting information about the conformational changes of the DNA in the lipoplexes, when the mass ratio L/D varies, is by circular dichroism, CD. Figure 5 shows the CD spectra of the CAL/DOPE/DNA lipoplexes for different α and L/D values. First, it was checked that the liposomes in the absence of DNA did not contribute to the spectra. All CD spectra were run taking as reference an aqueous buffer solution HEPES 10 mM, at pH = 7.4. Figure 5 shows the CD spectrum corresponding to pure DNA in aqueous buffered solution of HEPES 10 mM. This spectrum presents a negative band, at about 247 nm, due to the right-handed helicities of the polynucleotide, and a positive band, close to 280 nm, coming from the π–π stacking interactions between the bases. This spectrum is in agreement with that expected for the right-handed B form of the double-stranded ctDNA [51]. For all the CAL/DOPE/DNA lipoplexes investigated, a diminution in the positive band intensity upon increasing L/D was observed. The (Im_16_)_4_/DOPE/DNA lipoplexes could not be studied at higher L/D values because of solubility problems. This makes the comparison of the results obtained for this calixarene with those corresponding to the rest of the macrocycles more difficult.

The dependence of the positive band intensity on L/D could be explained considering the attractive electrostatic interactions between the DNA phosphate groups and the positively charged calixarenes within the lipoplexes. These interactions could cause the opening of the DNA double strand and conformational changes in the polynucleotide. An increment in L/D is accompanied by an increase in the amount of cationic calixarene in the lipoplexes. Therefore, it would be expected that the diminution in the positive band intensity was larger, for a given α value, the higher L/D is, as is observed.

The displacement of the inflection point (observed at 260 nm for pure DNA) towards higher wavelengths as well as the increase in the negative band intensity are usually related to the DNA denaturation and to DNA conformational changes [52,53]. Bombelli et al. found a similar dependence of the DNA spectrum in gemini surfactants/DOPE/DNA lipoplexes on L/D [54]. These authors proposed that the gemini surfactant/DNA interactions in the liposomes causes a conformational DNA change from a B form to a more condensed Ψ phase, where the polynucleotide molecules are partially inserted within an inverted hexagonal lipid rearrangement, which gives the DNA a certain spatial organization and a fixed directionality.

The displacement of the inflection point wavelength diminishes when α increases. This could be due to the effects of the presence of DOPE on the DNA conformation, as was pointed out by Marty et al. [55] in different liposome formulations. It is also observed in Figure 4 that the wavelength displacement is lower for (Im_12_)_4_ than for (TEAC_12_)_4_ (14 nm vs. 6 nm, respectively). Both calixarenes have the same hydrophobic tail length. This observation was explained considering that the imidazolinium groups were intercalated, at least partially, between the DNA base pairs, this stabilizing the B form of the DNA [56]. At this point it is worth noting that there are not enough experimental results for (Im_16_)_4_ that permit the comparison with the other CAL.

**Figure 6:**
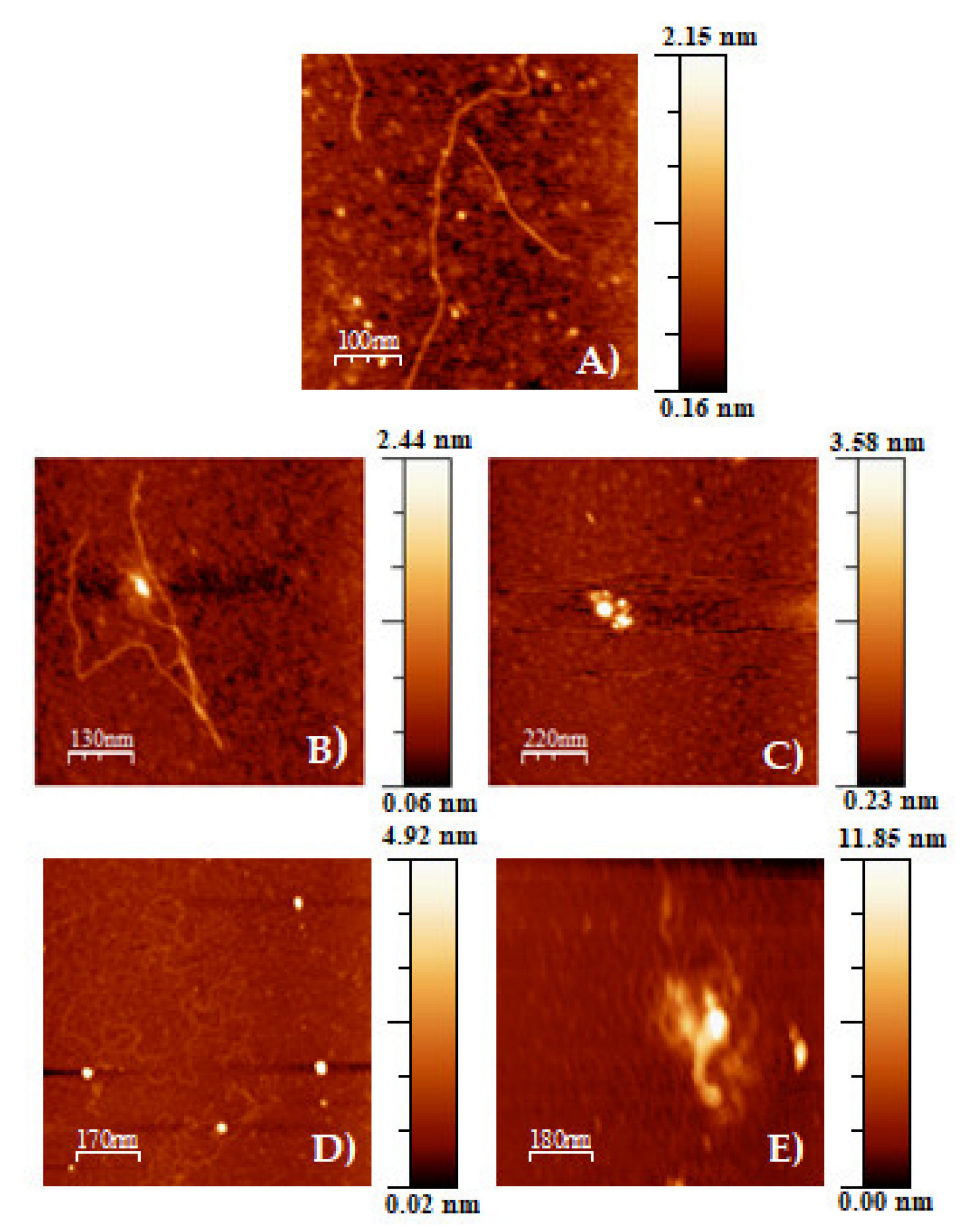
AFM topographic images of CAL/DOPE/DNA lipoplexes in buffered solutions, 10 mM HEPES (pH = 7.4), adsorbed on APTES modified mica surface. (**A**) Pure DNA; (**B**) (Im_3_Im_16_)_2_ α = 0.2 and L/D = 1; (**C**) (Im_3_Im_16_)_2_ α = 0.2 and L/D = 7; (**D**) (TEAC_12_)_4_ α = 0.2 and L/D = 1; (**E**) (TEAC_12_)_4_ α = 0.2 and L/D = 7.

The circular dichroism results seem to indicate that the lipoplex formation results in DNA conformational changes. In order to support this hypothesis, atomic force microscopy measurements, AFM, were carried out. Figure 6 shows the AFM images corresponding to pure DNA (Figure 6A), together with those of CAL/DOPE/DNA lipoplexes of different compositions, for two of the calixarenes investigated (Figure 6B–E). In the absence of liposomes, the pure DNA presents an elongated form (see Figure 6A). For (Im_3_Im_16_)_2_/DOPE/DNA and (TEAC_12_)_4_/DOPE/DNA lipoplexes at α = 0.2 for L/D = 1 (Figure 6B,D), which is lower than (L/D)_Φ_, some globular structures are observed, and the length of the DNA seems to be somewhat shorter, although no substantial conformational variations are observed. Yan et al. [57] explained the formation of the globular structures, linked across DNA strands, on the basis of an increased bending of the DNA double helix, this leading to the formation and stabilization of intramolecular loops. The separation of the double DNA strand into single strands, due to electrostatic attractions between the DNA and the cationic calixarenes, favors this process. For L/D = 7, (L/D) > (L/D)_Φ_, the number of globular structures present in the images increases. However, a full condensation of the polynucleotide is not reached (Figure 6C,E) since some elongated fragments of DNA are still observed. These experimental observations are in agreement with the CD spectra.

**Figure 7:**
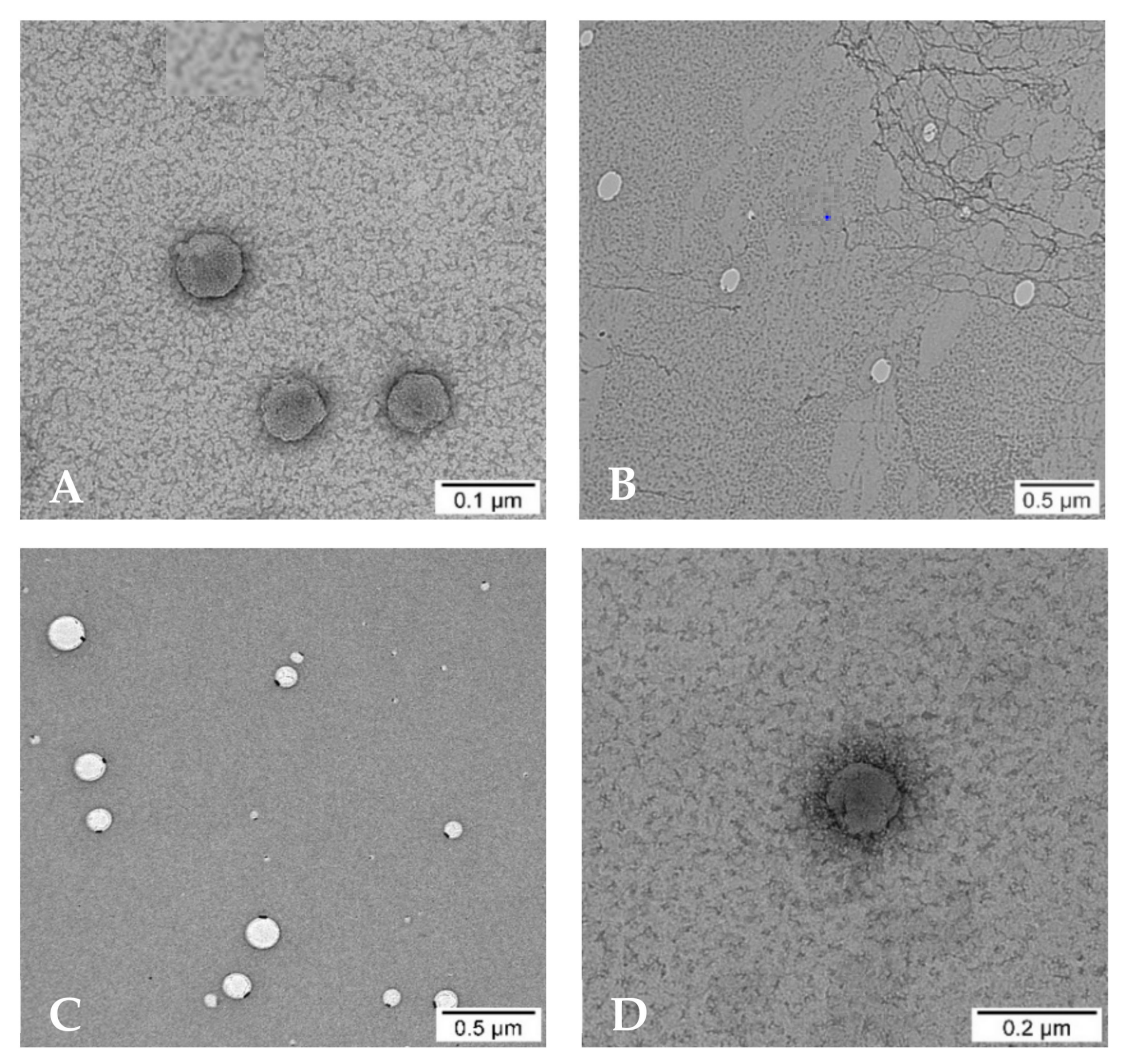
TEM images of the following systems: (**A**) (TEAC_12_)_4_/DOPE liposomes with α = 0.3; (**B**) (Im_12_)_4_/DOPE liposomes with α = 0.3; (**C**) (TEAC_12_)_4_/DOPE/DNA lipoplexes with α = 0.3 and L/D = 5; (**D**) (Im_12_)_4_/DOPE/DNA lipoplexes with α = 0.3 and L/D = 5.

With the goal of visualizing the morphology of the CAL/DOPE liposomes and CAL/DOPE/DNA lipoplexes, transmission electron microscopy measurements were carried out. Figure 7 shows the TEM images obtained for some of the systems investigated. One can see that a spherical morphology is observed for both the liposomes and the lipoplexes studied. The molar ratio α = 0.3 was chosen because Figure 1 shows that, for the two calixarenes studied, the minimum size is found close to this molar ratio value. It is interesting to indicate that (TEAC_12_)_4_/DOPE and (Im_12_)_4_/DOPE liposomes are the less cytotoxic among the calixarene-based liposomes investigated. For the mass ratio L/D = 5, the charge inversion of the polynucleotide is complete for (TEAC_12_)_4_/DOPE/DNA and (Im_12_)_4_/DOPE/DNA lipoplexes, at α = 0.3 (see Figure 3). Figure 7 shows that the two lipolexes not only are spherical, but their size is in the order of a few hundred nanometers. This is important in relation with the use of lipoplexes for gene delivery since it has been shown that a spherical morphology and a small size are two characteristics of the genetic material nanocarriers that favor transfection efficiency [49]. Besides, the liposome and lipoplex sizes measured using DLS (Figure 4) and TEM are in agreement, as one can see in Table 3.

### 3.3. Transfection Efficiency of CAL/DOPE/pDNA Lipoplexes

**Figure 8:**
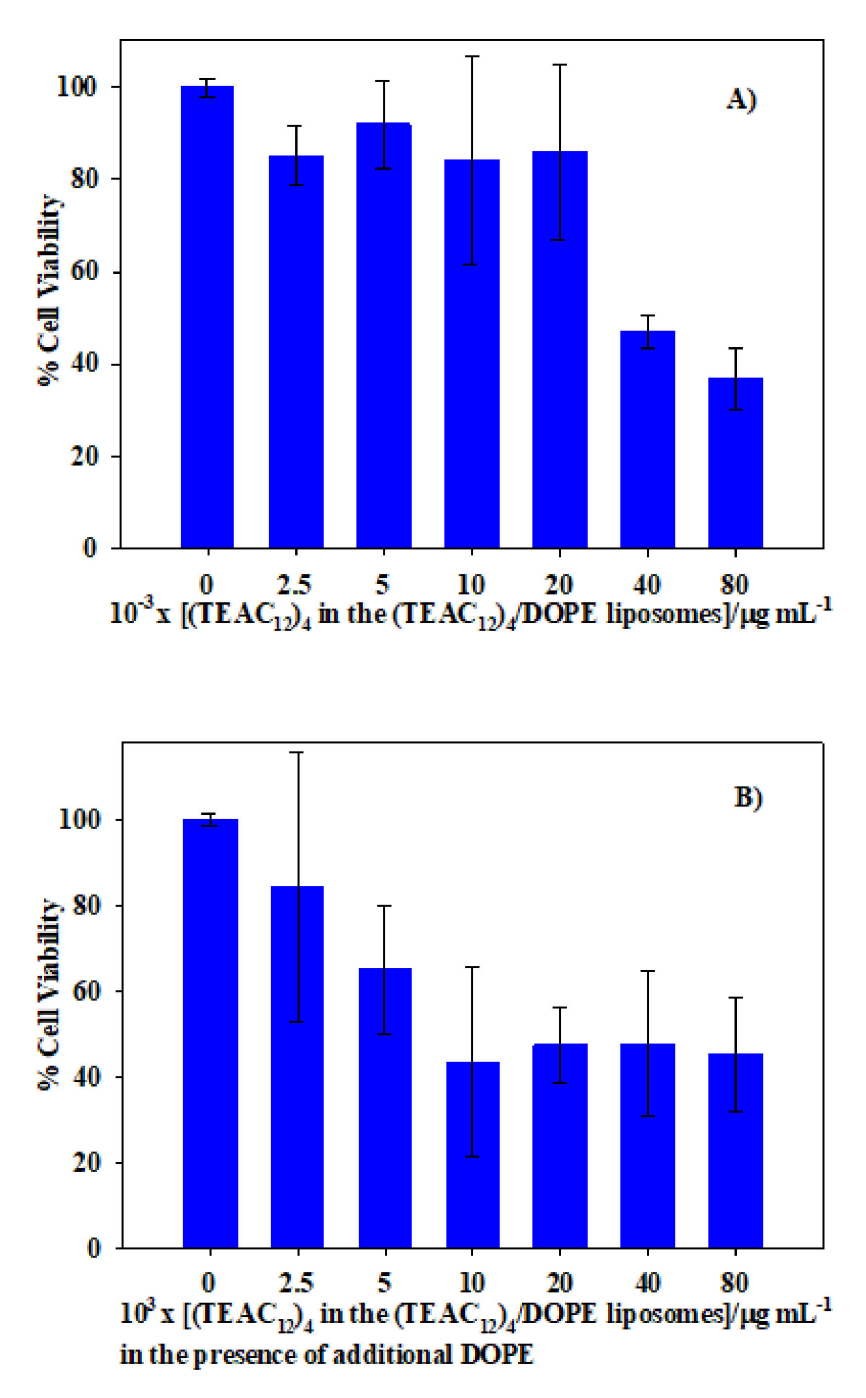
Dependence of the cell viability (%) of (TEAC_12_)_4_/DOPE liposomes in U2OS cancer cell line, at 48 h, on the concentration of (TEAC_12_)_4_ within the liposomes, for a constant cationic lipid molar ratio α = 0.3. The experiments were done in triplicate. (**A**) In the absence of additional DOPE; (**B**) in the presence of additional DOPE (+¼ of the DOPE amount present in the liposomes), which is not forming part of the (TEAC_12_)_4_/DOPE liposomes.

The transfection experiments were carried out for the lipoplexes containing the least cytotoxic calixarene: (TEAC_12_)_4_. Before carrying out these measurements, the cell viability of the liposomes for the human bone osteosarcoma epithelial cells U2OS using the MTT assay was carried out. This is the cell line used in the transfection experiments because it is considered an easy-to-transfer cell line. Figure 8 shows the results obtained. In this figure, the cytotoxicity of the (TEAC_12_)_4_/DOPE liposomes in the presence of additional DOPE (+1/4 of the DOPE amount present in the liposomes) is also presented. These systems were investigated because in the transfection experiments the addition of DOPE could make the delivery of genetic material more efficient [58]. Therefore, given that the transfection efficiency, TE, of the lipoplexes was studied in the presence of this phospholipid, the cell viability experiments in the presence of different cell lines for (TEAC_12_)_4_/DOPE liposomes + DOPE was also carried out.

One can see that, for α = 0.3, the presence of additional DOPE, which is not present in the liposomes, substantially diminishes the cell viability of (TEAC_12_)_4_/DOPE liposomes. In the absence of additional DOPE, the cell viability of the (TEAC_12_)_4_/DOPE liposomes is lower than 60% for concentrations of the cationic calixarene [(TEAC_12_)_4_] ≥ 20 μg mL^−1^. However, the addition of DOPE to the system caused the cell viability of the (TEAC_12_)_4_/DOPE liposomes to be lower than 60% for cationic calixarene concentrations [(TEAC_12_)_4_] > 5 μg mL^−1^. That is, keeping the cationic calixarene molar ratio α equal to 0.3, the cell viability substantially decreases when the [(TEAC_12_)_4_] within the liposomes increases if additional DOPE is present.

The transfection process of the plasmid pEGFP-C1 was carried out on the U2OS cells. The TE of the (TEAC_12_)_4_/DOPE/pEGFP-C1 lipoplexes, with α = 0.3, within a L/D range between 9 and 90, in the presence as well as in the absence of additional DOPE, was estimated. Expression of GFP is frequently used to follow transfection, and it does not require any additional manipulation of the sample since GFP is an intrinsically fluorescent protein. Therefore, its fluorescence can be readily measured directly. The lowest L/D value studied was 9, with the idea of assuring that the lipoplexes have a positive charge, a requirement to cross the cell membrane. On the other hand, L/D values higher than 90 were not investigated because this would mean a high amount of cationic calixarene present in the liposomes, this increasing cytotoxicity (see Figure 2). In regard to the introduction of additional DOPE in order to improve the TE, amounts of DOPE up to ¼ of that present in the lipoplexes were investigated. Higher additional phospholipid amounts were not studied to avoid a further increase in cytotoxicity (see Figure 8).

**Figure 9:**
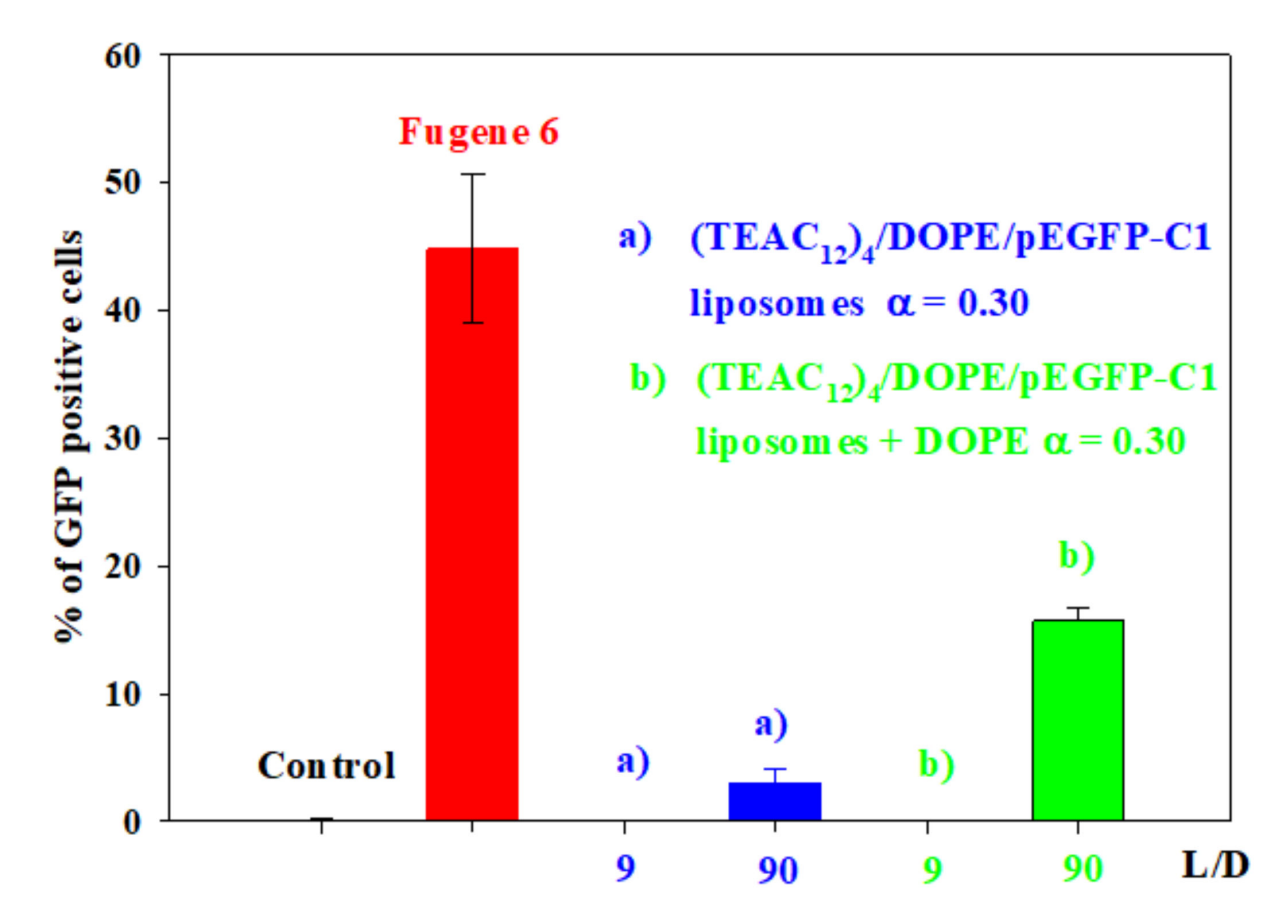
Percentage of GFP-positive cells after transfection with 3 μg of p-EGFP-C1, 24 h post transfection; 10,000 cells were analyzed per condition by flow cytometry (FACS). (a) (TEAC_12_)_4_/DOPE/pEGFP-C1 liposomes at α= 0.30; and (b) (TEAC_12_)_4_/DOPE/pEGFP-C1 liposomes + DOPE at α = 0.30.

Figure 9 shows that for the mass ratio L/D = 9 no transfection was observed in the absence as well as in the presence of additional DOPE. Negligible TE values were also found for L/D values lower than 90. For this reason, they are not shown in Figure 9. However, for L/D = 90 a low TE was observed, close to 3%. This TE is much lower than that of the FuGENE 6 reagent. When DOPE was added (¼ of the amount of phospholipid present in the lipoplexes), the TE for L/D = 90 increased up to 16%. For additional DOPE amounts lower than ¼ no changes in the TE for L/D=9 were observed. The increment in the TE caused by the addition of the helper lipid DOPE could be due to an increase in the stabilisation of the interactions cationic lipid/p-EGFP-C1 [59,60]. Besides, the addition of DOPE could also make the transfer of the genetic material in the context of endosomal escape more favorable, due to its fusogenic character [61].

**Figure 10:**
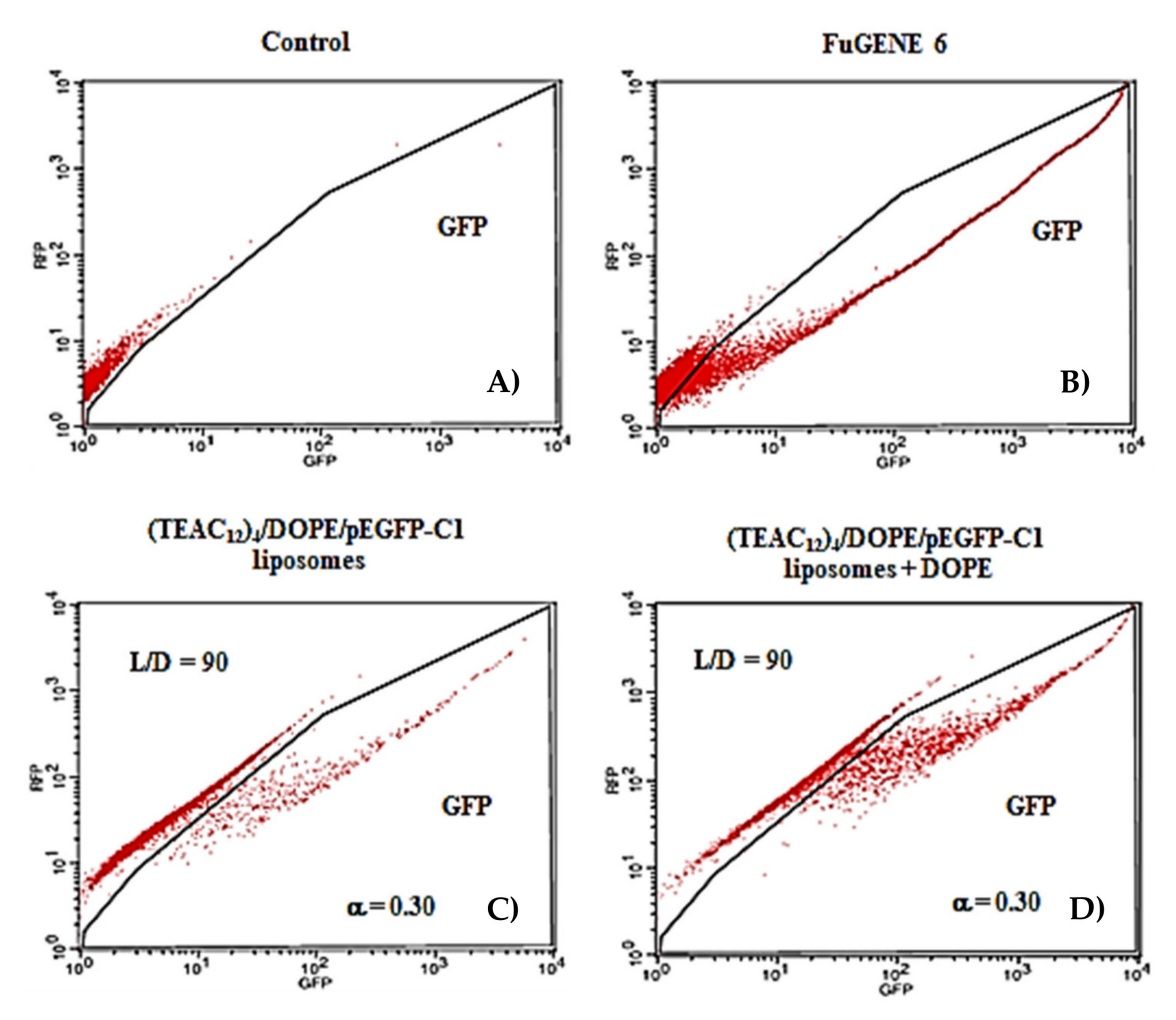
FACS analysis of cells transfected with 3 μg of p-EGFP-C1 for the different reagents. The charts show green fluorescent emission (X axis) vs. red fluorescent emission (Y axis) of 10,000 live cells analyzed per condition 24 h post-transfection. The GFP gate defines the area where cells with a clear increase in their green fluorescent emission are observed without a parallel increase in their red fluorescent emission. (**A**) Control; (**B**) FuGENE 6; (**C**) (TEAC_12_)_4_/DOPE/pEGFP-C1 liposomes at α = 0.30 for L/D = 90; and (**D**) (TEAC_12_)_4_/DOPE/pEGFP-C1 liposomes + DOPE at α = 0.30 for L/D = 90.

**Figure 11:**
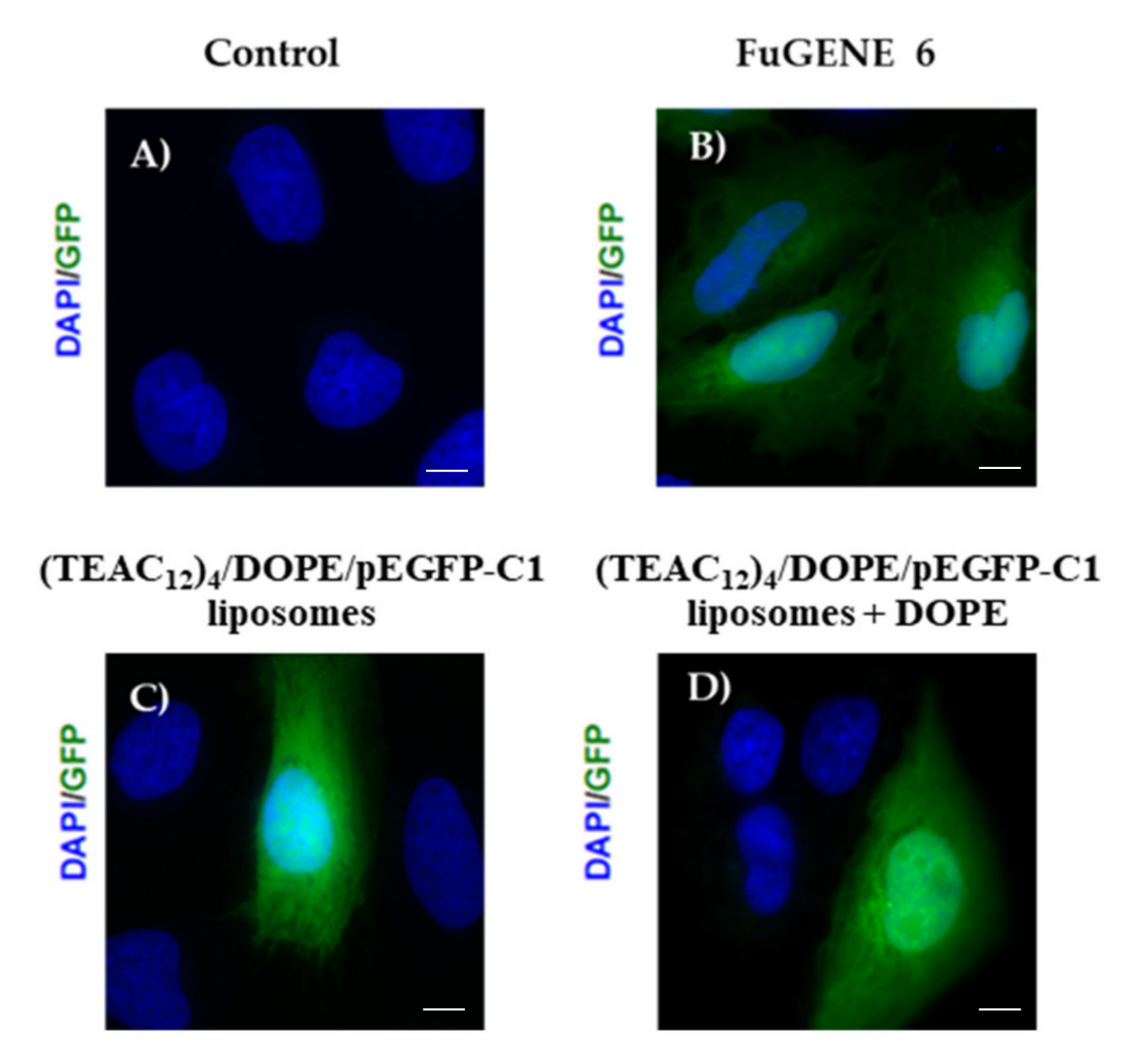
Representative images of GFP positive cells after transfection with 3 μg of pEPFG-C1. (**A**) Control; (**B**) FuGENE 6; (**C**) (TEAC_12_)_4_/DOPE/pEGFP-C1 lipoplexes at α = 0.3 and L/D = 90; (**D**) (TEAC_12_)_4_/DOPE/pEGFP-C1 lipoplexes at α = 0.3 and L/D = 90 in the presence of additional DOPE. Scale bar: 10 μm.

Figure 10 shows the FACS analyses of cells transfected with 3 µg of p-EGFP-C1 with the indicated reagents. Representative images of GFP-positive cells after transfection are shown in Figure 11.

### 3.4. Encapsulation of Doxorubicin

Doxorubicin (see Scheme 2), DOX, is an antineoplastic drug displaying a strong antitumoral activity against a wide spectrum of human cancers. It is used in the treatment of various lung, breast, or ovarian cancers. It is also used in chemotherapy for leukemia and lymphomas [62,63,64]. DOX intercalates between the base-pairs of the DNA, this resulting in the inhibition of the synthesis and transcription of the genetic material. The result is the blocking of the enzyme topoisomerase II, which hindered the division and growing of cells. Besides, the interactions DOX/DNA cause variations in the chromatin structure, which triggers apoptosis in cells [65]. In spite of the beneficial DOX activity, its clinical use is limited by its side effects. Gastrointestinal toxicity, stomatitis, myelosuppression, or cardiotoxicity are some of the most frequent side effects caused by the treatment with DOX [66,67,68].

The study of the encapsulation of drugs within nanocarriers is of great interest because it could permit the transportation of the drug towards its therapeutic target but, simultaneously, diminishing the drug side effects. One of the most frequently used nanocarriers are liposomes, particularly in the case of doxorubicin [69,70,71]. In fact, there is a commercial liposome preparation for DOX administration in chemotherapy called Doxil^®^ [72]. Other types of nanovehicles have also been used to administer doxorubicin [73,74,75,76]. In this work, the (TEAC_12_)_4_/DOPE liposomes were used to study the encapsulation and the release of doxorubicin. These liposomes were chosen because of the low cytotoxicity they present, which is one of the requirements of nanocarriers for biomedical applications.

**Figure 12:**
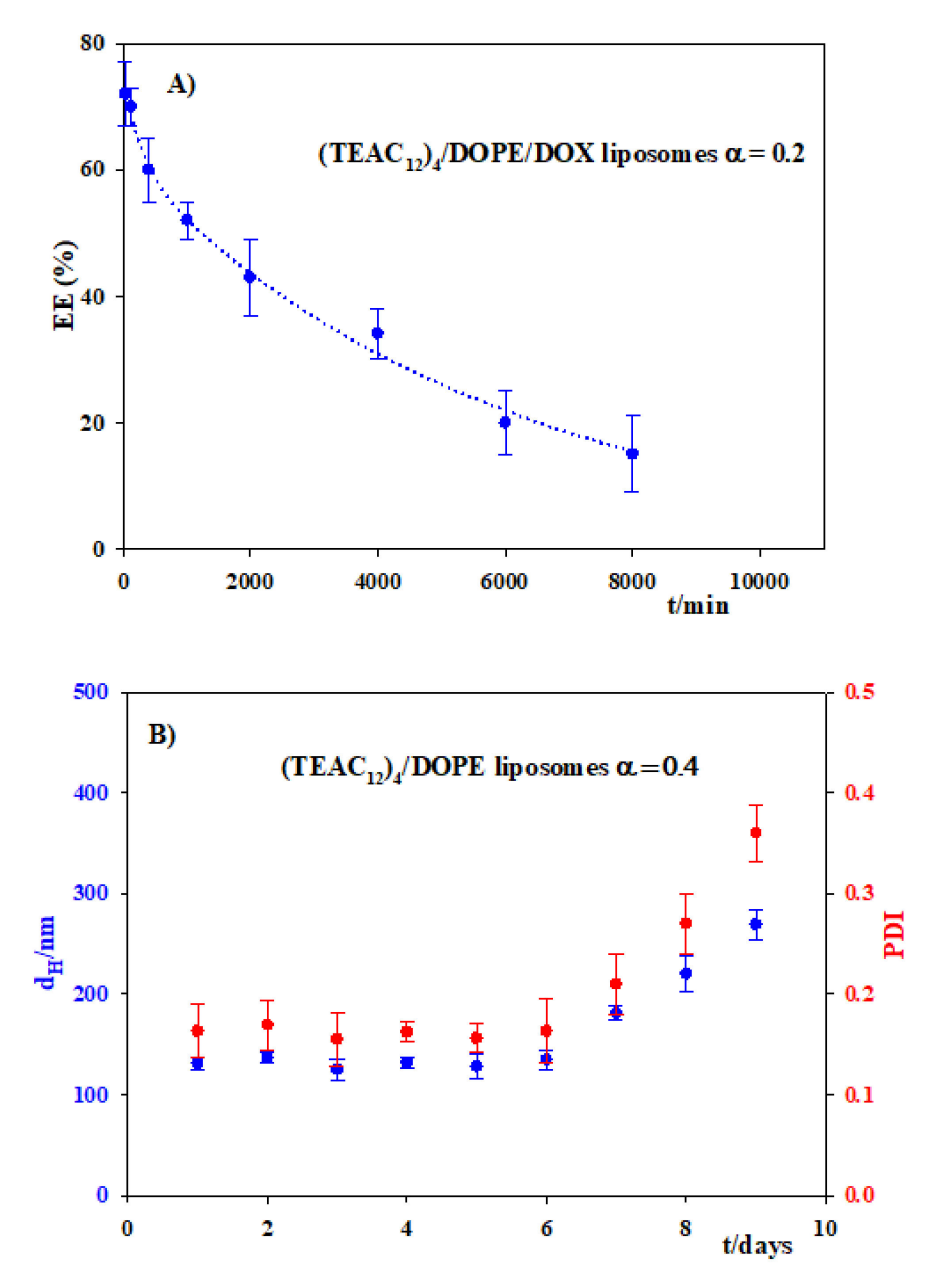
Variation of the hydrodynamic diameter, d_H_, and of the polydispersity, PDI, with time for (TEAC_12_)_4_/DOPE liposomes at 310 K. (**A**) α = 0.2; (**B**) α = 0.4.

Before studying the encapsulation of doxorubicin within the calixarene-based liposomes, the stability of these nanostructures was investigated. Stability was followed by DLS measurements, through the dependence of the hydrodynamic diameter, d_H_, and the polydispersity, PDI, on time, at 310 K (simulating the human body temperature). Figure 12 shows the results. One can see that the liposomes were stable during approximately 6 days. After that time the size as well as the PDI increased, this indicating that the system was not stable for longer times. The results observed in Figure 12 could be explained by the fragmentation of the lipid membrane of the liposomes due to the hydrolytic decomposition of the phospholipid molecules. As a consequence, their structure will vary, and an increment in the surface of the liposome membranes can occur, this causing an increase in both the hydrodynamic diameter and the polydispersity.

The encapsulation of doxorubicin within the (TEAC_12_)_4_/DOPE liposomes was done during the hydration process of the lipid bilayer (thin lipid film method). A DOX buffered solution (HEPES 10 mM, pH = 7.4) was added to the dry lipid bilayer. Afterwards, the vortex-sonication cycles were carried out, followed by the extrusion process. The final DOX concentration was 2 × 10^−4^ mol L^−1^ in all cases. In order to estimate the amount of DOX encapsulated, the drug-loaded liposomes were dialyzed (see Section 2.12). The doxorubicin concentration was determined by UV–visible spectroscopy measuring absorbance at 490 nm. The temperature was kept at 277 K in order to avoid doxorubicin degradation. The results obtained are summarized in Table 4.

Table 4 shows that for the two molar fractions α investigated, the encapsulation efficiency was high. EE% increased when α augmented. This experimental observation could be explained by considering the interactions between (TEAC_12_)_4_ and doxorubicin, which were investigated in a previous study [30]. An increase in the amount of the antineoplastic drug in the liposome, an increase in α, will favor these interactions, this leading to an increment in the encapsulation efficiency. The loading capacity also followed the same trend as EE%, and the explanation is similar to that given above.

The size and the polydispersity, PDI, of the (TEAC_12_)_4_/DOPE liposomes with and without loaded DOX were compared. The measurements were done by DLS, and the data are listed in Table 5. One can see that the hydrodynamic diameter as well as the PDI of the liposomes were similar in the absence and in the presence of doxorubicin.

**Figure 13:**
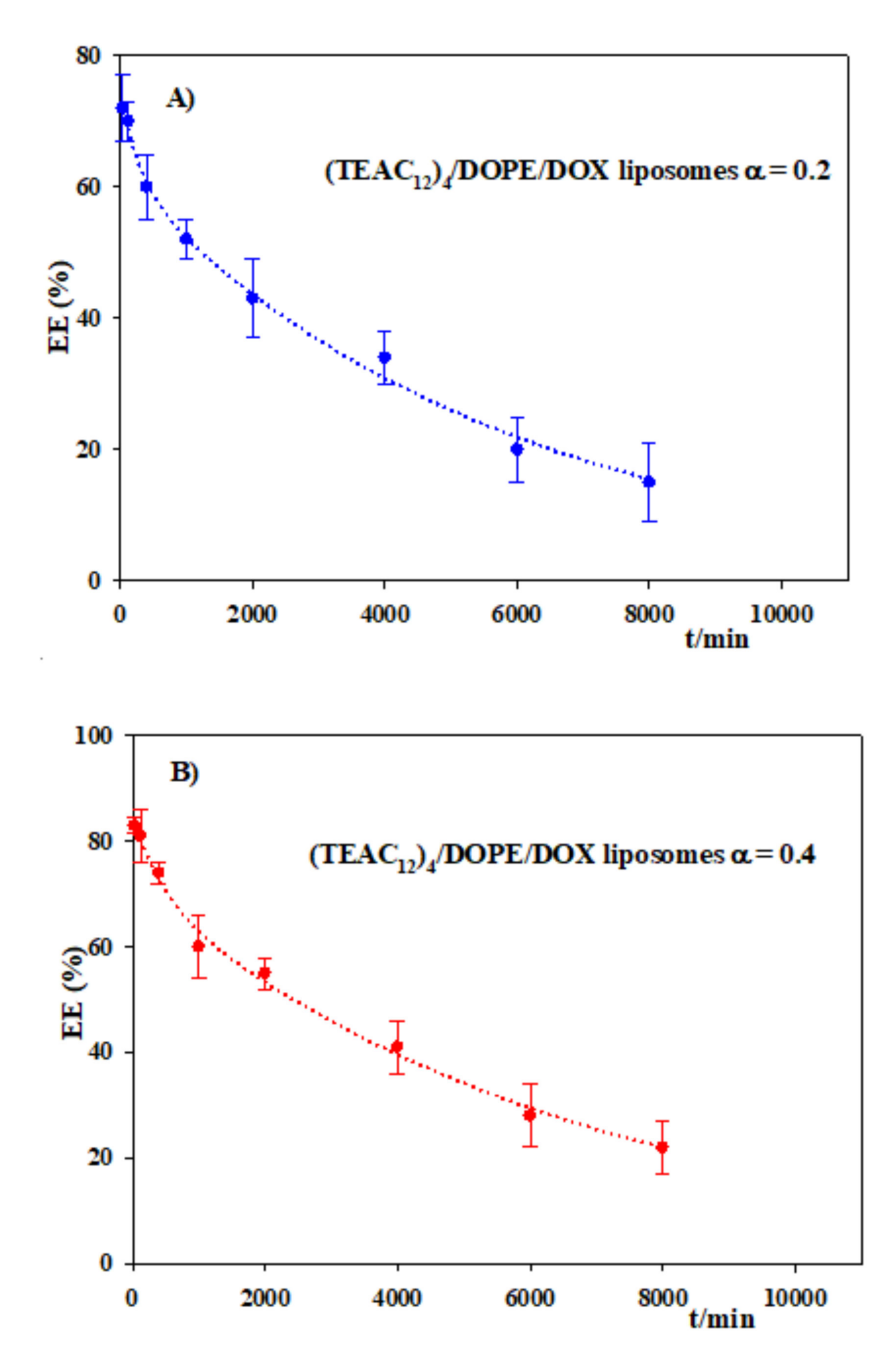
Release of doxorubicin from DOX loaded (TEAC_12_)_4_/DOPE liposomes at 310 K. (**A**) α = 0.2; (**B**) α = 0.4. The values are the average of three independent experiments.

When a drug is loaded in a nanocarrier, it is important to study the release time of the drug. The method used to investigate the release was described in the Experimental section, and it was carried out also at 310 K, in order to mimic the human body temperature. Figure 13 shows the variations of EE% against time for doxorubicin loaded (TEAC_12_)_4_/DOPE liposomes with molar fractions 0.2 and 0.4. The concentration of doxorubicin was estimated measuring the absorbance at 490 nm. From the variations of EE% with time, it is possible to deduce that the release follows a pseudo-first-order kinetics.

The following kinetic rate constants were estimated: 1.9 × 10^−4^ min^−1^ and 1.6 × 10^−5^ min^−1^ for α = 0.2 and α = 0.4, respectively. Figure 13 shows that not all the doxorubicin was released from the liposomes. This could be explained by considering that, once the liposomes are fragmented, CAL molecules free from the lipid bilayer will be present in the solution. (TEAC_12_)_4_ can form different aggregates at low CAL concentrations, and DOX could be bound to them [30]. This hypothesis is in agreement with the results shown in Figure 13, since less doxorubicin was released in the case of α = 0.4 than of α = 0.2. An increase in the molar ratio α means an increment in the amount of calixarene present in the liposomes. Therefore, once the liposomes are broken, there will be a larger number of free CAL molecules to associate with the doxorubicin and, consequently, the amount of antineoplastic drug released from the liposomes will be lower.

The results obtained indicate that (TEAC_12_)_4_/DOPE liposomes can be used as non-cytotoxic nanocarriers for the antineoplastic drug doxorubicin. Besides, even when liposomes are fragmented, the DOPE as well as the (TEAC_12_)_4_ molecules are non-cytotoxic [30]. The half-lives of the release were 2.5 and 3 days for α = 0.2 and α = 0.4, respectively, this pointing out that the side effects of the doxorubicin could be diminished due to a controlled release of the drug encapsulated as compared to the use of the naked drug.

## 4. Conclusions

In this work the formation of calixarene-based liposomes, CAL/DOPE, was investigated. Calixarenes with hydrophobic chains of different length attached to their lower rim were considered. The nature of the hydrophilic head present in their upper rim was also changed. The phospholipid DOPE was used, together with the cationic calixarene, for forming the lipid bilayer of the liposomes. The liposomes were characterized using several techniques. TEM images showed their spherical morphology. Cell viability experiments permitted the estimation of the cytotoxicity of the CAL/DOPE liposomes of different compositions, the results showing that the (TEAC_12_)_4_ liposomes are the least cytotoxic.

The formation and characterization of CAL/DOPE/DNA lipoplexes of different compositions was investigated and the nanostructures characterized. They have a spherical geometry and a size on the order of a few hundred nanometers. Subsequently, transfection experiments were carried out only for the least cytotoxic calixarene, (TEAC_12_)_4_. Results showed that some of these (TEAC_12_)_4_/DOPE/p-EFGP-C1 lipoplexes can transfect, although the transfection efficiency, TE, is low. However, the presence of an additional amount of DOPE substantially increases the TE. This could be explained considering that the presence of DOPE can stabilize the interactions between the cationic lipid and the plasmidic DNA. Besides, the addition of DOPE could also make the transfer of the genetic material in the context of endosomal escape more favorable, due to its fusogenic character.

The antineoplastic agent doxorubicin was encapsulated in the (TEAC_12_)_4_/DOPE liposomes with high encapsulation efficiencies. The liposomes were stable for close to 6 days, at 310 K (the human body temperature). The drug release was studied, and the results showed that the liposomes can be utilized for a controlled release of the drug. Their use could suppose a diminution in its side effects.

The results obtained also show that the calixarene-based liposomes seem to be better nanocarriers, for both nucleic acids and doxorubicin, than their aggregates (micelles and vesicles), formed by the naked calixarenes.

Future investigations will be oriented to the design and preparation of new non-cytotoxic amphiphilic calixarenes with the goal of using the CAL/DOPE/DNA lipoplexes as nanocarriers for the delivery of genetic materials, with a high transfection efficiency. The CAL/DOPE liposomes can also be checked as nanovehicles for different drugs.

## Figures and Tables

**Table 1:** Composition (mole ratio) of the CAL/DOPE liposomes prepared.

α	(TEAC_12_)_4_	(Im_12_)_4_	(Im_3_Im_16_)_2_	(Im_16_)_4_
0.1	-	-	1:9	1:9
0.2	1:4	1:4	1:4	1:4
0.3	1:2.33	1:2.33	1:2.33	1:2.33
0.4	1:5	1:5	1:5	-
0.45	1:1.22	1:1.22	-	-
0.5	1:1	1:1	1:1	-

Mole ratio is expressed with respect to the n_CAL_.

**Table 2:** Theoretical and experimental (L/D)_Φ_ values.

(L/D)_Φ_								
α	(TEAC_12_)_4_		(Im_12_)_4_		(Im_16_Im_3_)_2_		(Im_16_)_4_	
0.10	-		-		6.6 ^a^	6.6 ^b^	6.9 ^a^	6.9 ^b^
0.20	3.8 ^a^	3.6 ^b^	3.7 ^a^	3.6 ^b^	3.6 ^a^	3.7 ^b^	3.9 ^a^	3.9 ^b^
0.30	2.8 ^a^	2.6 ^b^	2.7 ^a^	2.6 ^b^	2.6 ^a^	2.6 ^b^	2.9 ^a^	2.9 ^b^
0.40	2.3 ^a^	2.2 ^b^	2.2 ^a^	2.0 ^b^	2.1 ^a^	2.1 ^b^	-	-
0.45	2.1 ^a^	1.8 ^b^	2.0 ^a^	1.8 ^b^	-	-	-	-
0.50	2.0 ^a^	1.8 ^b^	1.9 ^a^	1.7 ^b^	1.8 ^a^	1.8 ^b^	-	-

^a^ Theoretical values; ^b^ Experimental values.

**Table 3:** Sizes of various liposomes and lipoplexes, with a molar ratio α = 0.3, measured by dynamic light scattering, DLS, and electronic transmission microscopy.

	Hydrodynamic Diameter (DLS)/nm		Diameter (TEM)/nm	
	(TEAC_12_)_4_	(Im_12_)_4_	(TEAC_12_)_4_	(Im_12_)_4_
**Liposomes**	147 ± 4	157 ± 4	130 ± 32	140 ± 40
**Lipoplexes (L/D = 5)**	183 ± 9	276 ± 8	183 ± 39	223 ± 47

**Table 4:** Encapsulation efficiency, EE%, and loading capacity, LC, of doxorubicin in the (TEAC_12_)_4_/DOPE liposomes. T = 277 K. Three independent experiments were carried out for each system studied.

α	EE%	LC%
0.2	72 ± 5	1.85 ± 0.18
0.4	83 ± 1	4.21 ± 0.09

**Table 5:** Hydrodynamic diameter, d_H_, and polydispersity, PDI, of (TEAC_12_)_4_/DOPE liposomes and doxorubicin loaded (TEAC_12_)_4_/DOPE liposomes. T = 310 K. The results are the average of three independent experiments.

α	d_H_/nm ^a^	PDI ^a^	d_H_/nm ^b^	PDI ^b^
0.2	200 ± 7	0.23 ± 0.02	186 ± 6	0.18 ± 0.02
0.4	131 ± 6	0.163 ± 0.012	134 ± 6	0.22 ± 0.03

^a^ (TEAC_12_)_4_/DOPE liposomes. ^b^ DOX loaded (TEAC_12_)_4_/DOPE liposomes.

## Data Availability

Not applicable.

## Supplementary Materials

The following are available online at https://www.mdpi.com/article/10.3390/pharmaceutics13081250/s1, Deduction of Equation (5); Figure S1: Electrophoretic mobility shift assay on an agarose gel (1%) for CAL/DOPE/DNA lipoplexes; Figure S2: Dependence of the relative zeta potential, (ζ/ζ_o_), and of the hydrodynamic diameter, d_H_, of CAL/DOPE/DNA lipoplexes on L/D for α = 0.2. T = 303.0.1 ± 0.1 K.
