# Supplementary material for: Multivalent Calixarene-Based Liposomes as Platforms for Gene and Drug Delivery
Source: Pharmaceutics. 2021 Aug 12;13(8):1250. doi: 10.3390/pharmaceutics13081250 (PMC8398082; doi:10.3390/pharmaceutics13081250)
Supplement: Supplementary file 1 [file pharmaceutics-13-01250-s001.zip › pharmaceutics-1327426-supplementary.pdf]

pharmaceutics

Supplementary Material

Multivalent Calixarene-Based Liposomes as Platforms for Gene and Drug Delivery

José Antonio Lebrón [1], Manuel López-López [2], Clara B. García-Calderón [3], Ivan V. Rosado [3], Fernando R. Balestra [4,5], Pablo Huertas [4,5], Roman V. Rodik [6], Vitaly I. Kalchenko [6], Eva Bernal [1], María Luisa Moyá [1,*] Pilar López-Cornejo [1,*] and Francisco J. Ostos [1,*]

1 Department of Physical Chemistry, Faculty of Chemistry, University of Seville, C/ Profesor García González 1, 41012 Seville, Spain; jlebron@us.es (J.A.L.); evabernal@us.es (E.B.)
2 Department of Chemical Engineering, Physical Chemistry and Materials Science, Faculty of Experimental Sciences, University of Huelva, Campus de El Carmen, Avda. de las Fuerzas Armadas s/n, 21071 Huelva, Spain; manuel.lopez@diq.uhu.es
3 Institute of Biomedicine of Seville (IBiS), University Hospital Virgen del Rocío/CSIC/University of Seville, Avda. Manuel Siurot s/n, 41013 Seville, Spain; claragarcia@us.es (C.B.G.-C.); ivrosado@us.es (I.V.R.)
4 Department of Genetics, Faculty of Biology, University of Seville, C/ Profesor García González 1, 41012 Seville, Spain; fernando.balestra@cabimer.es (F.R.B.); phuertas@us.es (P.H.)
5 Andalusian Center of Molecular Biology and Regenerative Medicine (CABIMER), University of Seville-CSIC-University Pablo de Olavide, Avda. Américo Vespucio 24, 41092 Seville, Spain; fernando.balestra@cabimer.es (F.R.B.); phuertas@us.es (P.H.)
6 Institute of Organic Chemistry, National Academy of Science of Ukraine, 02660 Murmanska str. 5, Kiev, Ukraine; dmso@ukr.net (R.V.R.); vik@ioch.kiev.ua (V.I.K.)
* Correspondence: moya@us.es (M.L.M.); pcornejo@us.es (P.L.C.); fostos@us.es (F.J.O.); Tel.: +34-954557175 (M.L.M.)

1. Deduction of Equation (5)[1]

The mass ratio corresponding to neutral liposomes can be written as:

$$\left(\frac{L}{D}\right)_{\phi} = \left(\frac{L^{+} + L^{0}}{D}\right)_{\phi} \tag{1}$$

The effective charge of a liposome is defined as:

$$Z_{eff} = \frac{n_{+}}{n_{-}} = \left(\frac{q_{L^{+}}^{+}}{q_{ADN}^{-}}\right) \times \left(\frac{\left(\frac{L^{+}}{M_{L}^{+}}\right)}{\left(\frac{D}{M_{bp}}\right)}\right) \tag{2}$$

When Z_{eff}= 1, the ADN mass (D) can be written:

$$D = \left(\frac{q_{L^{+}}^{+}}{q_{ADN}^{-}}\right) \times \left(\frac{\left(\frac{L^{+}}{M_{L}^{+}}\right)}{\left(\frac{1}{M_{bp}}\right)}\right) \tag{3}$$

Equation (4) was obtained by considering Equations (1) and (3):

$$\left(\frac{L}{D}\right)_{\phi} = \frac{(L^{+} + L^{0})}{\left(\frac{q_{L^{+}}^{+}}{q_{ADN}^{-}}\right) \times \left(\frac{\left(\frac{L^{+}}{M_{L}^{+}}\right)}{\left(\frac{1}{M_{bp}}\right)}\right)} = \frac{q_{ADN}^{-}}{q_{L^{+}}^{+}} \times \frac{M_{L}^{+}}{M_{bp}} \times \left(\frac{L^{+} + L^{0}}{L^{+}}\right) = \frac{q_{ADN}^{-}}{q_{L^{+}}^{+}} \times \frac{M_{L}^{+}}{M_{bp}} \times \frac{(n^{+}M_{L}^{+} + n^{0}M_{L}^{0})}{n^{+}M_{L}^{+}} \tag{4}$$

If one multiplies by $[(n^{+} + n^{0})/(n^{+} + n^{0})]$, and introduces the molar fration α, Equation (5) is obtained. This equation relates $(L/D)_{\phi}$ and α:

$$\left(\frac{L}{D}\right)_{\phi} = \frac{q_{ADN}^{-}}{q_{L^{+}}^{+}} \times \frac{M_{L}^{+}}{M_{bp}} \times \frac{(n^{+}M_{L}^{+} + n^{0}M_{L}^{0})}{(n^{+}+n^{0})} \times \frac{1}{\frac{n^{+}}{(n^{+}+n^{0})}} = \frac{q_{ADN}^{-}}{q_{L^{+}}^{+}} \times \frac{M_{L}^{+}}{M_{bp}} \times \frac{\alpha M_{L}^{+} + (1-\alpha)M_{L}^{0}}{\alpha} \tag{5}$$

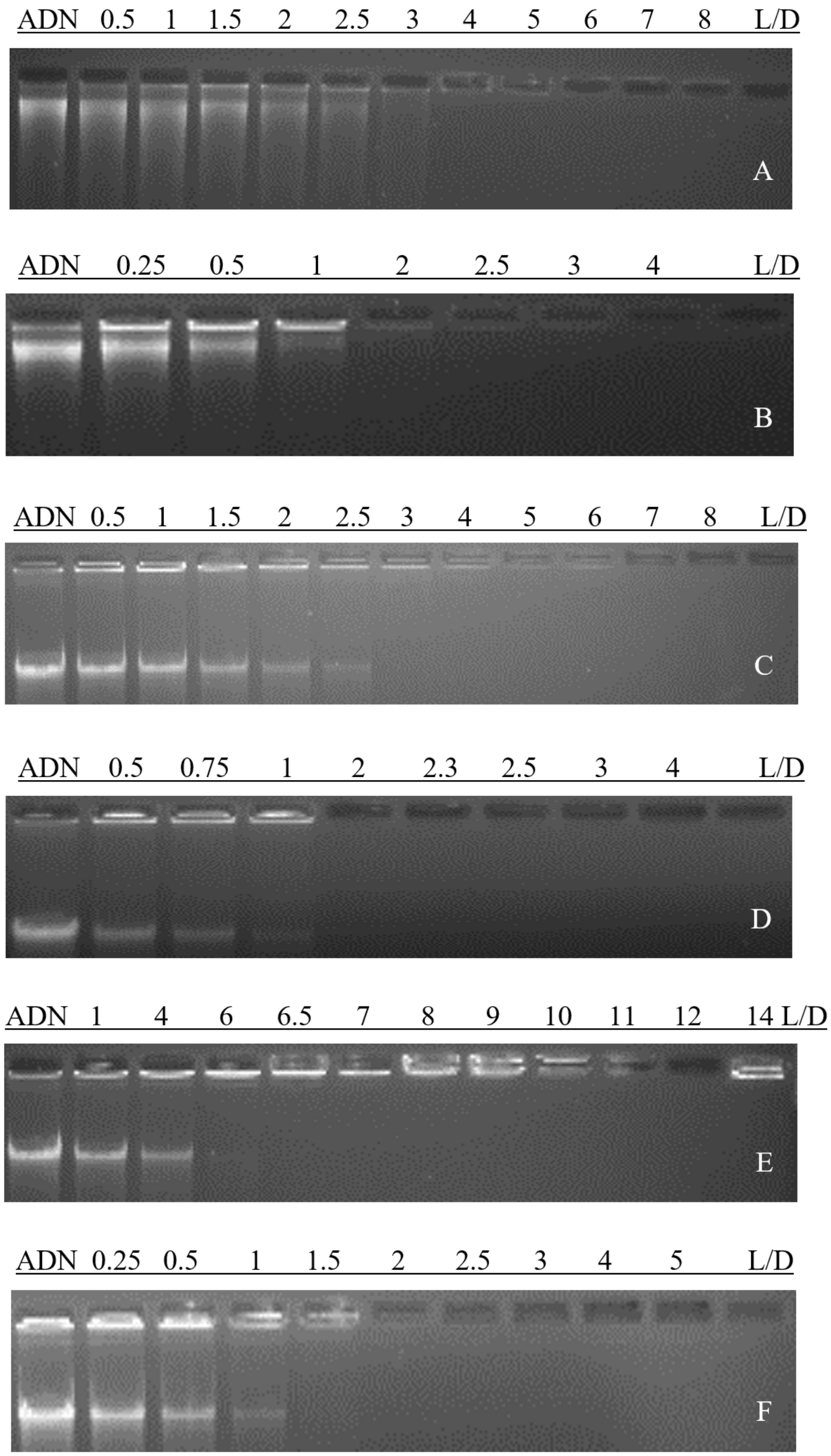
ADN 0.5 1 1.5 2 2.5 3 4 5 6 7 8 L/D
A
ADN 0.25 0.5 1 2 2.5 3 4 L/D
B
ADN 0.5 1 1.5 2 2.5 3 4 5 6 7 8 L/D
C
ADN 0.5 0.75 1 2 2.3 2.5 3 4 L/D
D
ADN 1 4 6 6.5 7 8 9 10 11 12 14 L/D
E
ADN 0.25 0.5 1 1.5 2 2.5 3 4 5 L/D
F

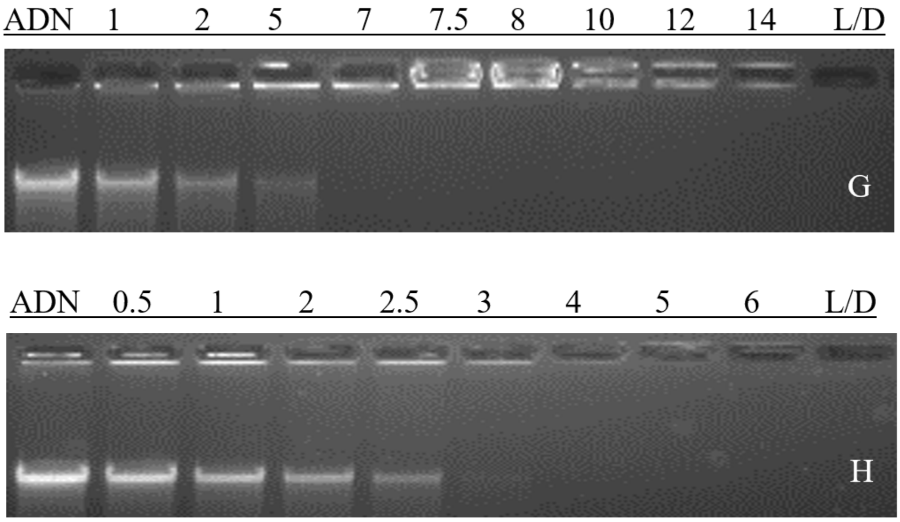

Figure S1. Electrophoretic mobility shift assay on an agarose gel (1%) for CAL/DOPE/DNA lipoplexes at different α values, varying the mass ratio L/D. Experiments were run in TAE buffer (40 mM tris-acetate, 1 mM EDTA) and stained with Red Safe. Electrophoresis was performed at 90 V until completion. $(TEAC_{12})_4$: (**A**) $\alpha = 0.2$ y (**B**) $\alpha = 0.5$; $(Im_{12})_4$: (**C**) $\alpha = 0.2$ y (**D**) $\alpha = 0.5$; $(Im_{16}Im_3)_2$: (**E**) $\alpha = 0.1$ y (**F**) $\alpha = 0.5$, and $(Im_{16})_4$: (**G**) $\alpha = 0.1$ y (**H**) $\alpha = 0.3$.

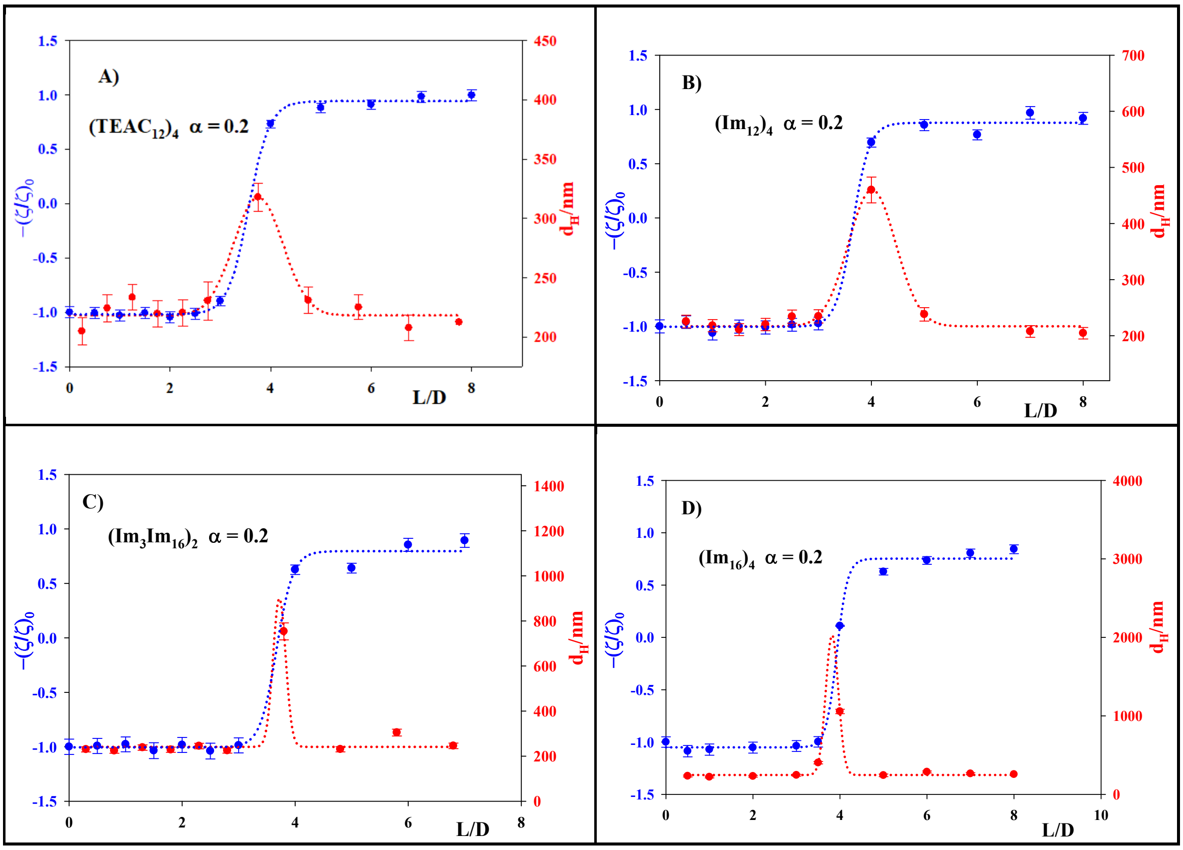

Figure S2. Dependence of the relative zeta potential,-(ζ/ζ_o), and of the hydrodynamic diameter, d_H, of CAL/DOPE/DNA lipoplexes on L/D for $\alpha = 0.2$. T = 303.0.1 ± 0.1 K. (**A**) $(TEAC_{12})_4$; (**B**)$(Im_{12})_4$; (**C**) $(Im_3Im_{16})_2$; (**D**) $(Im_{16})_4$.

Reference:

1. M. Múñoz-Úbeda, S.K. Misra, A.L. Barrán-Berdon, C. Aicart-Ramos, M.B. Sierra, J. Biswas, P. Kondaiah, E. Junquera, S. Bhattacharya, E. Aicart, *J. Am. Chem. Soc.* **2011**, *133*, 18014-18017. https://doi.org/10.1021/ja204693f.
